# Extraction of metallic aluminum from fluidized bed and grate incineration bottom ash

**DOI:** 10.1177/0734242X261444139

**Published:** 2026-05-02

**Authors:** Simon Mika, Julia Mühl, Jakob Lederer

**Affiliations:** 1 Christian Doppler Laboratory for Design and Assessment of an Efficient, Recycling-Based Circular Economy, Institute of Chemical, Environmental and Bioscience Engineering, TU Wien, Vienna, Austria

**Keywords:** aluminum, extraction, recovery, recycling, incineration, bottom ash, dry-wet IBA treatment, municipal solid waste incineration, MSWI, fluidized bed, grate incineration

## Abstract

The extraction of metallic aluminum from incineration bottom ash (IBA) has twofold implications: to obtain aluminum for the recycling industry and to reduce the content in the treated mineral fraction for its utilization as aggregate in concrete. This study used IBAs from fluidized bed (FB) and grate (G) municipal solid waste incineration for two full-scale experiments, investigating the flows of metallic aluminum in a dry-wet IBA treatment process. Metallic aluminum >4 mm was determined by hand sorting, while <4 mm was determined by the soda-attack method. Extraction rates into metal concentrates of 97% for FB-IBA and 78% for G-IBA were achieved, demonstrating the plant’s ability to treat both types of IBA. The elevated extraction rate for FB-IBA is attributable to the dry IBA discharge and the reduced bed temperature. These factors preclude the melting of aluminum, glass, and minerals, resulting in the presence of aluminum particles that are entirely liberated from the mineral phase of IBA, which is advantageous for eddy current separation. Consequently, low residual metallic aluminum contents in the treated mineral fraction (0–8 mm) of 0.5 wt-% in FB-IBA and 1.0 wt-% in G-IBA were determined. Nevertheless, to draw conclusions on the usability in concrete, it is imperative to implement continuous monitoring of the residual metallic aluminum content to substantiate these values, given their reliance on sound plant operation as well as on the initial IBA composition, which knowingly exhibits significant fluctuations.

## Graphical abstract

**Visual Abstract:**
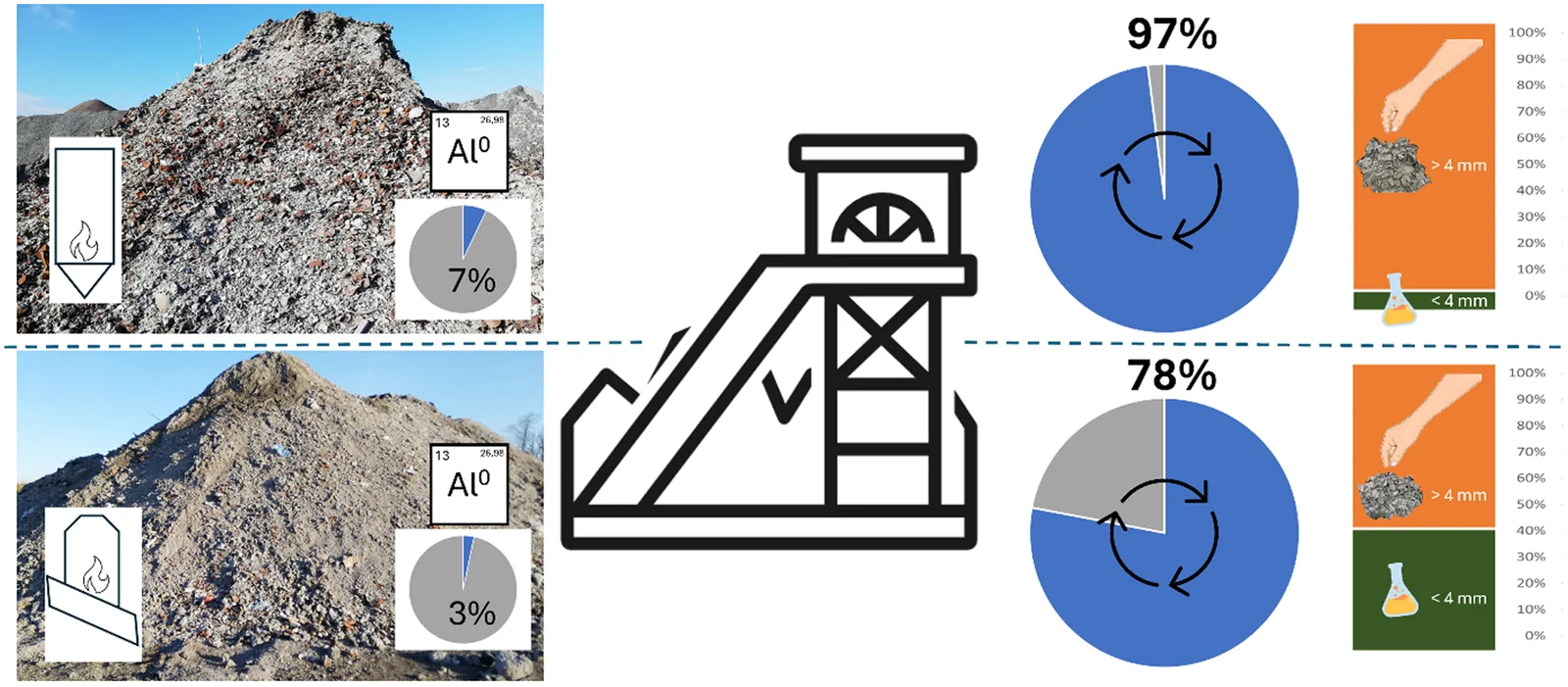
This is a visual representation of the abstract.

## Introduction

Municipal solid waste (MSW) incineration (MSWI) is an integral part of many MSW management systems worldwide (Khan et al., 2022). It enables the generation of energy from non-recyclable waste, as well as the recovery of materials from incineration residues (Brunner and Morf, 2025). Incineration bottom ash (IBA), which accounts for the bulk MSWI residues, contains ferrous and non-ferrous metals that are recovered in most countries (Šyc et al., 2020). This reduces the environmental burden of IBA landfills and, when provided to the recycling industry, the global warming potential (GWP) of metal production is reduced (Hagelüken et al., 2016; Mehr et al., 2021). Metallic aluminum is particularly significant in this regard, as substantial savings in GWP can be achieved (Allegrini et al., 2015). Growing attention is also being paid to the mineral fraction for use as industrial manufactured aggregate (IMAG; Lederer et al., 2025; Mühl et al., 2024; Verbinnen et al., 2017; Zou et al., 2025). A key parameter for utilizing the mineral fraction in concrete is the residual metallic aluminum content (Sun et al., 2024; van der Wegen et al., 2013) due to its chemical reaction with the alkalinity of the cement, leading to the formation of hydrogen, which increases the pore volume and reduces the strength and durability of the concrete (Chen et al., 2023; Müller and Rübner, 2006; Saikia et al., 2008; Tang et al., 2016). Therefore, extracting metallic aluminum from IBA has two implications: recovery for recycling and decreasing the content in the IBA mineral fraction, enabling its utilization as IMAG in concrete. In both cases, the extraction rate of metallic aluminum into target (metal concentrates) and non-target fractions is a crucial indicator of a successful IBA treatment.

This study investigates the extraction of metallic aluminum on an industrial scale in a dry-wet IBA treatment plant located in Austria. Two full-scale treatment experiments were carried out using two different types of MSWI-IBA. The first experiment was conducted with a dry-discharged IBA from a stationary fluidized bed (FB) incinerator (FB-IBA) incinerating pre-treated MSW. It was selected because industrial-scale treatment of FB-IBA is rarely described in the literature (Mika et al., 2025b; Mühl et al., 2024), and this study presents a complete balance of metallic aluminum for the first time. The second experiment used a wet-discharged IBA from a grate (G) incinerator (G-IBA) incinerating untreated MSW. It represents the most widely produced type of IBA globally and was chosen for the experiments due to its high relevance to IBA management. The objectives of this study were to determine the fate and flows of metallic aluminum of FB-IBA and G-IBA in an industrial-scale dry-wet IBA treatment plant. Furthermore, the extraction rates of metallic aluminum, as well as the residual metallic aluminum content in the treated mineral fraction, were assessed and discussed.

## Materials and methods

### Origin of the fluidized bed (FB-) and grate incineration bottom ash (G-IBA)

Both waste incinerators are located in Vienna, Austria, and incinerate exclusively MSW generated in Vienna. The fluidized bed incinerator (FB-I) is a stationary (bubbling) type, incinerating approximately 120,000 tonnes annually, of which 95% is mechanically pre-treated mixed MSW from households (mMSW) and 5% sewage sludge. The mechanical treatment includes shredding, screening, and ferrous metal separation; however, non-ferrous metal separation is not currently applied. The fuel obtained from this mechanical treatment has a particle size of 0–250 mm that is fed to the FB-I (Blasenbauer et al., 2023). The bed temperature is intentionally kept below 660°C to prevent sintering of ash and bed material, while the freeboard temperature (gas zone) above the bed reaches 930°C (Kirnbauer and Kraft, 2017). The FB-IBA is dry-discharged and screened for the recovery of bed material, which is periodically renewed (Krobath and Thomé-Kozmiensky, 2004). The grate incinerator (G-I) is a forward-acting reciprocating grate type that incinerates 250,000 tonne year^−1^, comprising 70% mMSW from households, 15% shredded bulky waste, and 15% other medical and industrial waste (Mika et al., 2025a). The fuel in G-I is exposed to temperatures up to 1000°C, but the fuel bed experiences considerable temperature fluctuations (Leckner and Lind, 2020). The IBA is wet-discharged, followed by magnetic separation of ferrous metals after the G-IBA is removed from the quench water tank (Huber et al., 2020). Pictures of the raw FB-IBA and G-IBA before treatment are shown in the Supplemental Section S2.1.

### Dry-wet incineration bottom ash (IBA) treatment plant

The dry-wet IBA treatment plant used in the experiment was already described in the scientific literature (Mika et al., 2025b; Mühl et al., 2024; Pfandl et al., 2020). Therefore, only a short summary of the process is provided hereafter, while a process scheme is presented in the Supplemental Section S2.2. The dry treatment includes screening, manual sorting of material >50 mm targeting metals and unburnt material, crushing of mineral agglomerates >50 mm as well as crushing of recirculated metal-depleted mineral material to below 8 mm, magnetic separation of ferrous metals, sensor-based sorting for glass recovery, and a two-stage eddy current separation (ECS) for non-ferrous metal recovery. The first ECS (ECS1) operates in a two-split-configuration, obtaining a concentrate (ECS1 concentrate) and a pre-concentrate of non-ferrous metals (ECS1 middlings). The ECS1 concentrate is a non-ferrous metal fraction with a high aluminum content and low mineral impurities, while the ECS1 middlings pre-concentrate contains different non-ferrous metals and a high content of mineral impurities. The second ECS operates recovery-driven with an ordinary one-split-configuration, yielding a non-ferrous metal fraction (ECS2) with low metal content. It is positioned at the end of the treatment process and is responsible for ensuring a low residual metallic aluminum content in the treated mineral fraction.

**Figure 1.**
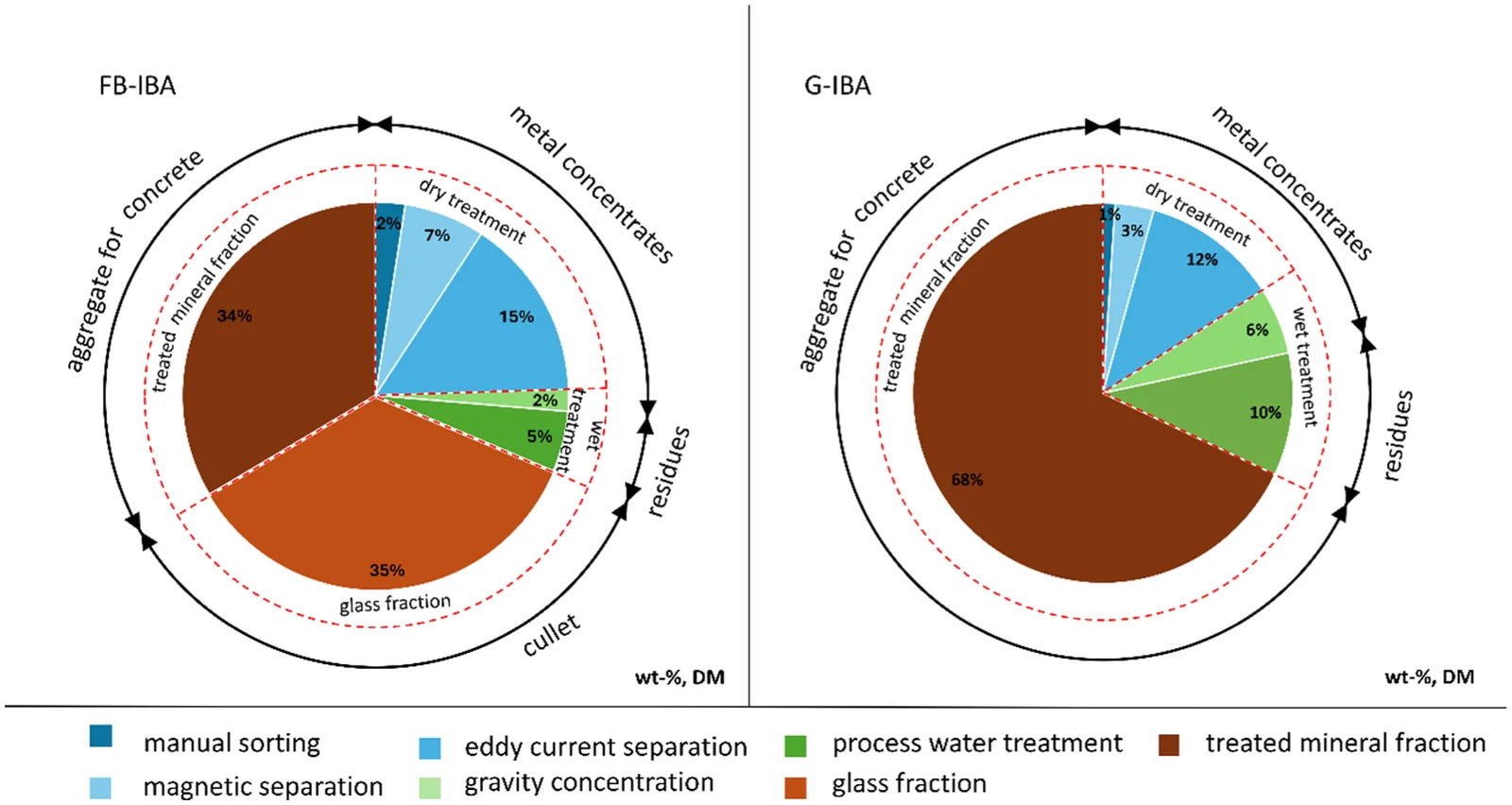
Percentual distribution of outputs obtained from the processing of fluidized bed incineration bottom ash (FB-IBA, left) and grate incineration bottom ash (G-IBA, right) in the dry-wet IBA treatment plant (Mühl et al., 2024).

The wet treatment includes a jig, centrifugal concentrators, a shaker table, and a hydrocyclone. Treatment of process water involves removing swimming material and sedimentation of sludge, followed by centrifugation of the process water before it is recirculated into the jig. Glass is only recovered from FB-IBA due to the superior quality and yield that can only be achieved from FB-IBA, while the quality of the mineral fraction (0–8 mm) of both, FB-IBA and G-IBA, is improved by the treatment due to the reduction of soluble salts, residual metal particles, and heavy metals (Mühl et al., 2023). The distribution of material flows in the experiments, comprising all 16 output flows of the treatment plant, was published in Mühl et al. (2024). Figure 1 presents these material flows for FB-IBA and G-IBA with the output flows allocated to respective treatment units (inner circle), to dry or wet treatment (middle circle), and to secondary raw materials or residues (outer circle). While the metal concentrates and the glass fraction require further upgrading prior to recycling, the treated mineral fraction must be stored and aged before it can be used as IMAG in concrete. A detailed list of the individual output flows, including their allocations, is presented in the Supplemental Section S2.2.

### Treatment experiments, sampling, and preparation of laboratory samples

The IBA treatment experiments were described in detail in Mika et al. (2025b) and Mühl et al. (2024). Since the same samples were analyzed in this study, the experiments are only briefly described. Two experiments were conducted with 190 tonnes dry matter (DM) each, one using FB-IBA and the other using G-IBA, originating from the FB-I and G-I plant described in section “Origin of the fluidized bed and grate incineration bottom ash”. The dry-wet IBA treatment plant was operated at full scale, with each experiment lasting 8 hours. All output flows of the treatment plant were incrementally sampled from moving lots in at least 30 increments using tailor-made hand devices and a wheel loader to sample the IBA mineral fraction. After sampling, the primary samples of the output flows were subsampled by incremental shoveling to obtain laboratory samples for analysis. In each case, care was taken to exceed the minimum sampling mass, which was calculated in advance for each output flow (details were described in Mika et al. (2025b) and values are presented in the Supplemental Section S2.3 in Table T2. All output flows and the input to the plant were weighed as a total by the plant operator’s personnel, except for the mineral fraction, which was mathematically balanced afterwards. The water content was determined immediately after subsampling for all output flows. As described by Mika et al. (2025b), three groups of output flows were obtained, which required different sample preparation. A list of the exact sample preparation for each output flow is presented in the Supplemental Section S2.3 in Table T3. Equation (1) was used to calculate the metallic aluminum content for each output flow *j* (j = 1. . .16), where $c_{\mathrm{Al}}$ is the content of metallic aluminum in either the overall output flow *j* or within a respective particle size fraction and ω is the mass fraction of the respective particle size fraction.

(1) $$c_{\mathrm{Al},j}=\omega_{>4}\cdot c_{\mathrm{Al},>4}+\omega_{<4}\cdot c_{\mathrm{Al},<4}$$

The first group of output flows consisted only of metallic particles >4 mm and was characterized by hand sorting. The second group consisted of output flows with particle sizes <4 mm, containing metallic and mineral particles. The laboratory samples of this group were totally or fractionally milled in a disk mill (Essa LM201 from FLSmidth, Copenhagen, Denmark). The latter obtained fractions with particle sizes >0.5, 0.1–0.5, and <0.1 mm. All size classes of the milled samples were then chemically analyzed individually (see section “Determination of metallic aluminum contents”). The content of metallic aluminum in these samples was then calculated according to equation (2), where $c_{\mathrm{Al}}$ is the content of metallic aluminum, and ω is the mass fraction of the respective particle size fraction.

(2) $$\begin{array}{l} c_{\mathrm{Al},<4\mathrm{mm}}=\omega_{>0.5\mathrm{mm}}\cdot c_{\mathrm{Al},>0.5\mathrm{mm}}+\omega_{0.1-0.5\mathrm{mm}}\\ \;\;\;\;\;\;\cdot c_{\mathrm{Al},0.1-0.5\mathrm{mm}}+\omega_{<0.1\mathrm{mm}}\cdot c_{\mathrm{Al},<0.1\mathrm{mm}} \end{array}$$

The output flows of the third group consisted of metallic and mineral particles <4 and >4 mm. These were prepared differently for FB-IBA and G-IBA, since metallic particles had to be liberated from mineral agglomerates in G-IBA, while they occur entirely liberated from minerals in FB-IBA (Blasenbauer et al., 2023). Therefore, the samples from G-IBA were spread on a metal plate, crushed with a vibrating roller (BW 65S from BOMAG, Boppard Germany) and sieved at 4 mm, while the samples from FB-IBA could immediately be sieved at 4 mm. The material >4 mm was hand sorted, while that <4 mm was fractionally milled and chemically analyzed. The total content was then calculated according to equation (2).

### Determination of metallic aluminum contents

When sorted by hand, aluminum was identified by its grayish-silver color, diamagnetism, and softness when filed with a metal file. To differentiate between aluminum and zinc >4 mm, dense media separation with ferrosilicon was used (Mika et al., 2025b). Chemical analysis of particles <4 mm was done differently depending on the particle size fraction. The fraction >0.5 mm of fractionally milled samples consisted only of flattened metallic particles. The material was digested with a mixture of HCl and HNO_3_ in a beaker, filtered, and the filtrate was analyzed using Inductively Coupled Plasma Optical Emission Spectroscopy (ICP-OES) (Optima 8300 from PerkinElmer, Waltham, USA). The obtained aluminum content originated entirely from metallic aluminum. In contrast, the milling fractions below 0.5 mm were expected to contain both metallic and mineral-bound aluminum, which cannot be distinguished by ICP-OES. To determine only the metallic aluminum content, the soda-attack method (or water-displacement method), considered state-of-the-art in IBA characterization, was applied (Chen et al., 2024; López et al., 2015; Vateva and Laner, 2020). Thereby, a sample was suspended in NaOH, which reacts with metallic aluminum, resulting in hydrogen evolution. By measuring the volume of hydrogen generated, the initial amount of metallic aluminum in the sample can be calculated using the ideal gas law. A calcimeter (SCM1 Behrotest ^®^Calcimeter from behr Labor-Technik, Düsseldorf, Germany) was used for the analysis. Details are presented in the Supplemental Section S2.4.

Based on the principle of mass conservation, the metallic aluminum content in the overall IBAs was determined by reverse calculation (equation (3)). It represents a weighted sum and was calculated with the software STAN 2.7 (TU Wien/Inka Software, Vienna, Austria) using a simple MFA model (1 process with 1 input flow and 16 output flows; Cencic, 2016).

(3) $$c_{\mathrm{Al},\mathrm{input}}=\sum_{j=1}^{16}\omega_{j}\cdot c_{\mathrm{Al},j}$$

In equation (3), $c_{\mathrm{Al}}$ is the content of metallic aluminum, the index *j* (*j* = 1. . .16) represents an output flow, and $\omega_{j}$ is the mass fraction of that output flow in the overall IBA. The method has the advantage that the output flows of a treatment process are significantly more homogeneous than the input, and hence smaller sample sizes are required to achieve the same relative sampling error as when untreated IBAs are analyzed (Morf et al., 2013).

## Results and discussion

### Metallic aluminum determined in the treatment units of the dry-wet IBA treatment plant

**Figure 2.**
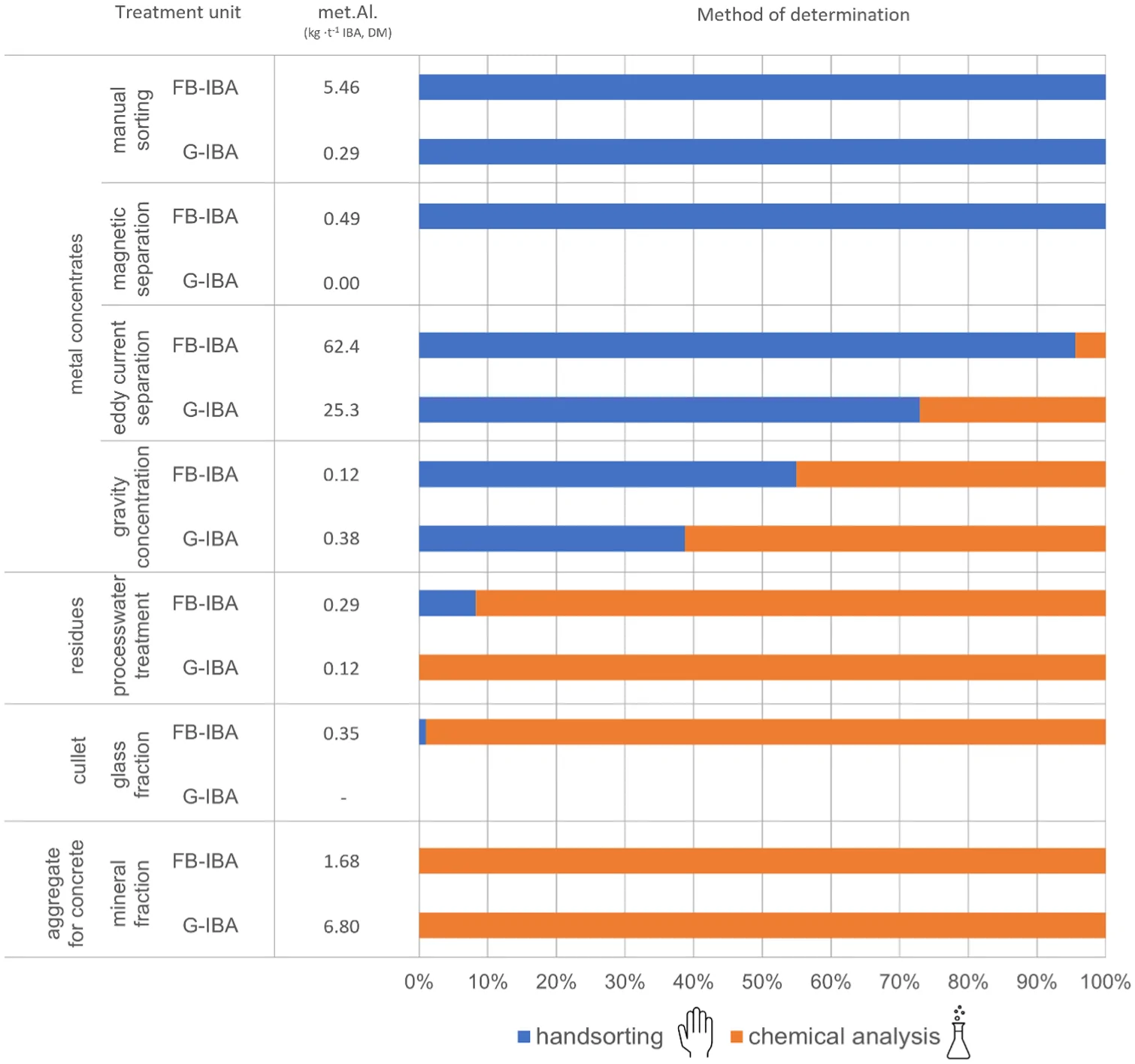
Metallic aluminum within the treatment units of the IBA treatment plant in kg·t^−1^ incineration bottom ash (IBA), dry matter (DM) and how much was determined by each method.

The amount of metallic aluminum determined within each treatment unit, expressed in kg·t^−1^ IBA on a DM basis, is presented in Figure 2 and discussed in the subsequent paragraphs. Additionally, it is shown how much of the total amount was determined by each method. A detailed list for each individual output flow is provided in the Supplemental Tables T4 and T5 in Section S3.1.

#### Target fractions for metallic aluminum (metal concentrates)

As shown in Figure 2, metallic aluminum in the treatment unit *manual sorting* yielded 5.46 kg·t^−1^ IBA, DM in FB-IBA treatment, whereas it yielded only 0.29 kg·t^−1^ IBA, DM in G-IBA treatment. Since only particles >50 mm were manually sorted, this indicates that FB-IBA contains more aluminum particles in this size class than G-IBA. A content of 0.49 kg·t^−1^ IBA, DM metallic aluminum, was determined in the treatment unit *magnetic separation* of FB-IBA, which consisted of composites with ferrous metals (e.g., spray cans with steel lid). Conversely, such composites were not detected in G-IBA, likely because most of the aluminum is molten in composites with ferrous metals, making intact composites less common and therefore the sample size analyzed was too small to detect them. Identification in both treatment units was done solely by hand sorting. ECS yielded 62.4 kg·t^−1^ IBA, DM from FB-IBA, of which 96% was determined by hand sorting and 4% by chemical analysis. From G-IBA, it yielded 25.3 kg·t^−1^ IBA, DM of which 73% was determined by hand sorting and 27% by chemical analysis. The share of metallic aluminum determined by chemical analysis was higher in G-IBA because the sample preparation required crushing and screening of the fraction to liberate metallic particles from mineral coating, which transferred some of these particles into the <4 mm fraction. Whether the particle size of these metallic particles was initially <4 mm fraction, or the impact of crushing shifted them into this fraction, could not be evaluated (see also section “Limitations”). *Gravity concentration* obtained 0.12 kg·t^−1^ IBA, DM from FB-IBA (55% determined by hand sorting, 45% by chemical analysis) and 0.38 kg·t^−1^ IBA, DM from G-IBA (39% determined by hand sorting, 61% by chemical analysis).

**Figure 3.**
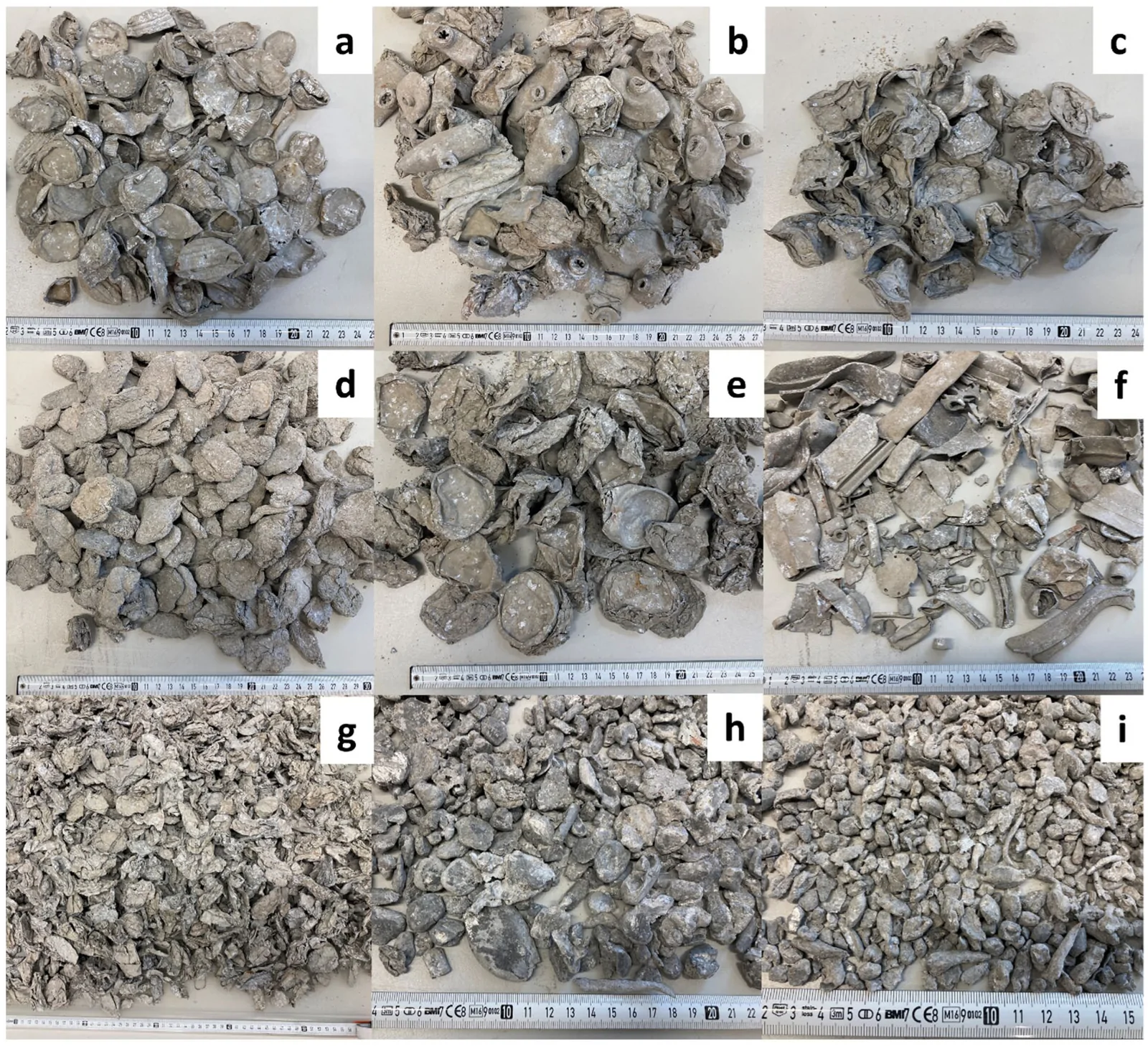
Metallic aluminum particles recovered by eddy current separation in the output flow“ECS1 concentrate” from fluidized bed incineration bottom ash (FB-IBA): bottle caps (a), food tubes (b), coffee capsules (c), household aluminum foil (d), used beverage cans (e), non-packaging products (f), crumbled up aluminum packaging products (g) and from grate incineration botton ash (G-IBA): lumps 8–50 mm (h), lumps 4–8 mm (i).

In Figure 3, some examples of metallic aluminum particles, which were contained in the ECS1 concentrate of FB- and G-IBA, are shown. Pictures of the original samples are shown in the Supplemental Section S3.1.1. The aluminum particles recovered from FB-IBA were obtained in a similar shape and wall thickness as initially present in MSW. Therefore, it was possible to distinguish between non-packaging and packaging products (Figure 3(a)– (f)), although many thin-walled packaging products were crumpled up from thermal and mechanical impacts (Figure 3(g)). A share of 67% packaging aluminum from the total metallic aluminum was found in FB-IBA, which is attributed to the MSW input of the FB-I, which is exclusively from households. Clearly, the aluminum was only exposed to a temperature below 660°C (the melting point of metallic aluminum). The same distinction was not possible for aluminum recovered from G-IBA, as it appeared mainly in lumps and nugget-like shapes, which is typical for a metal that was (partially) molten and solidified again (see Figure 3(h) and (i)). The same was described by Van Caneghem et al. (2019), when determining aluminium packaging in G-IBA.

The different appearances of recovered aluminum particles result from the varying conditions to which the waste is exposed in FB-I and G-I, as well as in the IBA discharge systems (López-Delgado et al., 2003). Metallic particles are fully liberated from minerals in FB-I because the non-combustible fraction of MSW is exposed to lower temperatures than it is on the grate and is cooled by air instead of water. Specifically, the temperature of the bed material in FB-I is maintained below 660°C to prevent sintering of ash, melting of glass, aluminum, and other non-ferrous metals, which leads to defluidization (De Gisi et al., 2018; Jones et al., 2013). In contrast, the high temperature on the grate leads to the (partial) melting of aluminum and other non-ferrous metals, and to the formation of mineral melt agglomerates (the so-called “slag”; Eusden et al., 1999). Additionally, quench agglomerates are formed when G-IBA is water-cooled in a wet-bottom ash discharger. Therefore, metallic particles are covered by a mineral layer in G-IBA (Wei et al., 2011). Photos of melt- and quench agglomerates from G-IBA are presented in the Supplemental Section S3.1.1.

#### Non-target fractions for metallic aluminum (glass and residues)

The glass fraction was only obtained from FB-IBA, revealing 0.35 kg·t^−1^ IBA, DM metallic aluminum (1% determined by hand sorting, 99% by chemical analysis). Although this amount was considered lost for recycling in this study, the glass fraction is in fact further upgraded to obtain *cullet* where the metallic aluminum might be (partially) recovered.

The metallic aluminum content of the entire treatment unit r*esidues* from G-IBA was determined by chemical analysis. In FB-IBA, the swimming material >16 mm was hand sorted, due to the presence of aluminum household foil, while the swimming material <16 mm and the sludge from sedimentation and centrifugation were chemically analyzed. Photos of the swimming material are presented in the Supplemental Section S3.1.2. In total, 0.21 kg·t^−1^ IBA, DM for FB-IBA and 0.12 kg·t^−1^ IBA, DM for G-IBA were found within *residues*. Since the residues are landfilled, this amount is lost for recycling.

#### Treated mineral fraction

As already mentioned, the metallic aluminum content within the treated mineral fraction is a key parameter for its use as IMAG in concrete. Amounts of 1.68 kg·t^−1^ IBA, DM in the treated mineral fraction of FB-IBA, and 6.8 kg·t^−1^ IBA, DM of G-IBA were determined by chemical analysis. According to equation (2) and the data provided by Mühl et al. (2024) on the metallic aluminum content >4 mm in the same experiments, it was possible to determine that 76% (FB-IBA) and 82% (G-IBA) of the metallic aluminum of the mineral fraction has a particle size <4 mm (the calculation is presented in the Supplemental Section S3.1.3).

In G-IBA, the content in the mineral fraction <4 mm can be explained by the generation of small metallic aluminum particles due to the melting of aluminum in G-I, which are then encapsulated in mineral agglomerates (Nithiya et al., 2018; Šyc et al., 2024). Given that metallic aluminum is not molten in the FB-I, an alternative explanation for FB-IBA must be posited. One such possibility is that thin-walled aluminum packaging products become brittle during thermal treatment and fracture easily when subjected to mechanical processing. This phenomenon was also observed in dry-discharged G-IBA, wherein a substantial proportion of metallic aluminum was found to be transferred to the dust fraction during material processing (Mehr et al., 2021; Skutan, 2023a).

### Comparison of the metallic aluminum content within industrial manufactured aggregate produced in other IBA treatment plants

In order to utilize the mineral fraction, it is a common practice to fractionate IBA and treat and utilize the material differently depending on the particle size (Sormunen and Rantsi, 2015; Vateva and Laner, 2020). A comparison of the metallic aluminum content within IMAG produced from G-IBA in different IBA treatment plants reveals that many plants produce aggregate only from the coarse mineral fraction >2 mm (Table 1). The comparison in Table 1 is limited to G-IBAs, because in the scientific literature, no other plant was identified that also produces aggregate from FB-IBA. Furthermore, with one exception, only dry-wet treatment plants were chosen, since they are designed to produce IMAG to be used in concrete. The one exception is a dry-treatment plant for dry-discharged G-IBA located in Switzerland, which was included because of its outstanding performance (85% aluminum extraction rate) although the treated mineral fraction is landfilled.

**Table 1.** Comparison of industrial manufactured aggregate (IMAG) produced from grate incineration bottom ash (G-IBA) in different treatment plants regarding metallic aluminum content and particle size fraction.

Location of treatment plant	Plant type	Particle size fraction of IMAG (mm)	Total content met.Al. (wt-%)	References
Austria (this plant)	Stationary (dry-wet)	4–8	0.2	Mühl et al. (2024)
		0–8	1.0	This study
Belgium	Stationary (dry-wet)	2–6	1.0	Joseph et al. (2020)
Germany	Mobile (dry-wet)	2–8	1.2	Vateva et al. (2025)
Netherlands	Stationary (dry-wet)	2–12	0.7	van der Wegen et al. (2013)
		4–11	0.6	Chen et al. (2024)
Switzerland	Stationary (dry)	2–12	0.6	Mehr et al. (2021)

In this study, a content of 1.0 wt-% within the 0–8 mm aggregate produced from dry-wet treatment of G-IBA was determined, while a content of 0.2 wt-% was previously reported for the 4–8 mm aggregate from G-IBA. For the investigated FB-IBA, a content of 0.5 wt-% within the 0–8 mm aggregate was determined, while a content of 0.1 wt-% in the 4–8 mm aggregate was previously reported (Table 2; Mühl et al., 2024). A study from Lederer et al. (2024) revealed that the dry-wet treatment plant described in this study is able to produce 2–8 mm aggregate made from other FB-IBAs, containing 0.1 wt-% metallic aluminum (stationary FB-I) and 0.01 wt-% (circulating FB-I; Table 2).

**Table 2.** Comparison of IMAG produced from fluidized bed incineration bottom ash (FB-IBA) of different fluidized bed incinerators (FB-I)in the same dry-wet treatment plant in Austria regarding particle size fraction and metallic aluminum content.

Location of treatment plant	FB-I (all located in Austria)	Particle size fraction of aggregate (mm)	met.Al. (wt-%)	References
Austria (this plant)	A (stationary type)	2–8	0.1	Lederer et al. (2024)
	B (circulating type)	2–8	0.01	Lederer et al. (2024)
	Same as in this study	4–8	0.1	Mühl et al. (2024)
	This study	0–8	0.5	This study

### Calculated total content of metallic aluminum in FB- and G-IBA

A total metallic aluminum content of 7.1 ± 0.6 wt-% in FB-IBA and 3.3 ± 0.4 wt-% in G-IBA was determined via reverse calculation (equation (3)). Similar metallic aluminum contents of 3.0–3.9 wt-% were previously reported for G-IBAs from Austria and Switzerland (Huber et al., 2020; Mehr et al., 2021), while lower contents of 0.6–2.2 wt-% were reported for G-IBAs from the Czech Republic, Denmark, Germany, and China (Allegrini et al., 2014; Šyc et al., 2018; Vateva and Laner, 2020; Xia et al., 2017). Regarding FB-IBA, Blasenbauer et al. (2023) reported a lower metallic aluminum content of 5.7 wt-% for the same FB-IBA than it was determined in this study. The reason for this is that the previous study was done in 2017, while the aluminum packaging content in mixed MSW increased from 2017 to 2022 (Mika et al., 2025a).

Mühl et al. (2024) determined a content of 7.0 wt-% and 2.9 wt-% of metallic aluminum >4 mm within the same FB-IBA and G-IBA by hand sorting of the particles >4 mm. This demonstrates that it is possible to determine most of the metallic aluminum by hand sorting. The deltas to what was obtained in this study can mainly be attributed to the amount <4 mm in the mineral fraction. The difference in total metallic aluminum content between the two types of IBAs is significant, and several reasons contributing to it must be addressed. A major influence is the amount of IBA that is produced relative to the input, which is 12 wt-% in FB-IBA and 20 wt-% in G-IBA (Blasenbauer et al., 2023), which means that the metals in FB-IBA are much more concentrated. Additionally the content of metallic aluminum within the fly ash is higher in FB-I than in G-I (Mika et al., 2025a). Therefore, the transfer coefficients of metallic aluminum are very different in both technologies.

### Metal extraction rate of metallic aluminum

**Figure 4.**
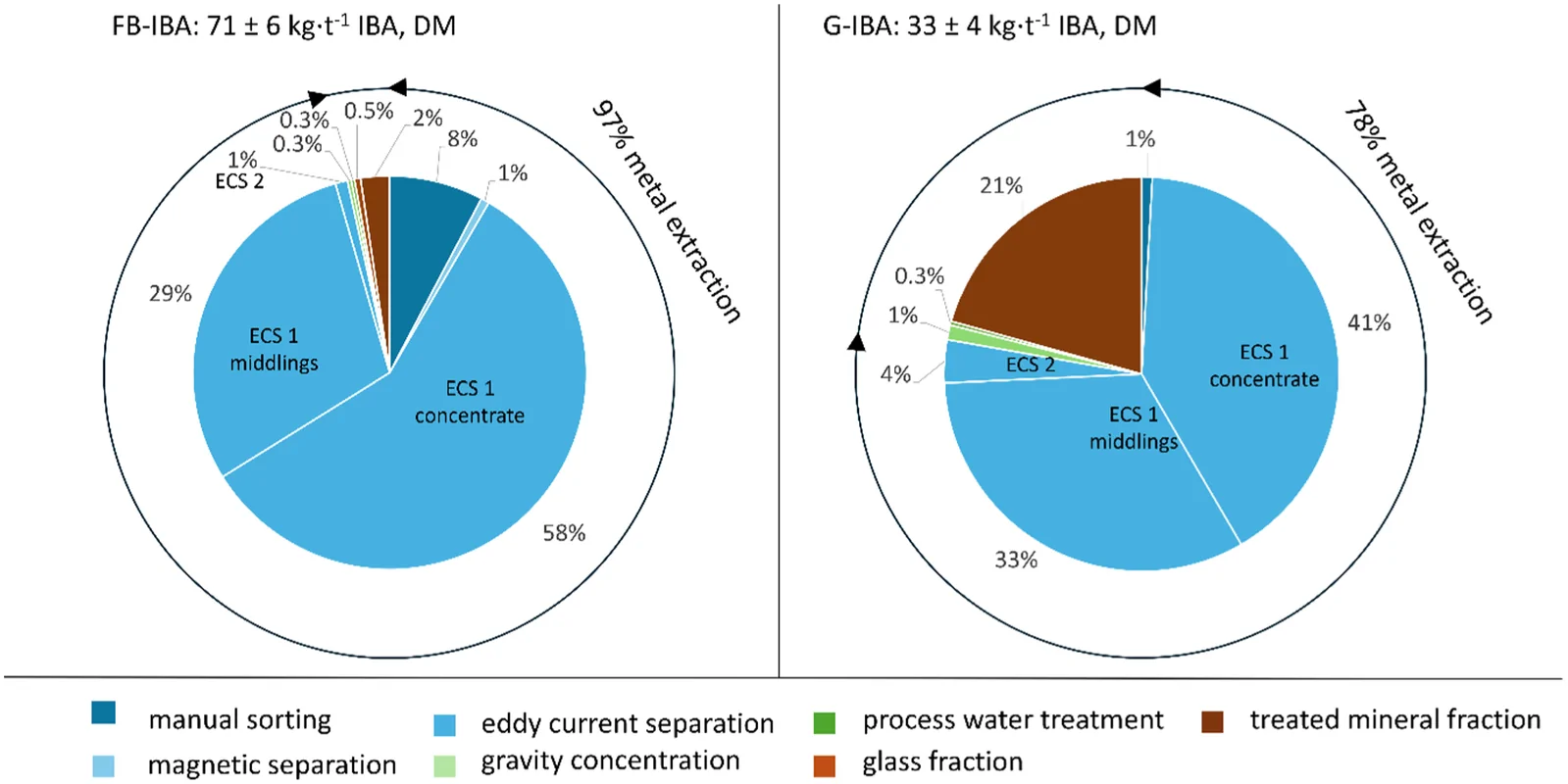
Metal extraction rates and distribution of the metallic aluminum contained in fluidized bed incineration bottom ash (FB-IBA, left) and grate incineration bottom ash (G-IBA, right) among the treatment units of the dry-wet IBA treatment plant, given in % of the total amount.

The distribution of metallic aluminum in the input among the treatment units of FB-IBA and G-IBA is presented in Figure 4. The data are presented in the Supplemental Section S3.1.

Considering only the extraction into metal concentrates, extraction rates were determined to be 97% for FB-IBA and 78% for G-IBA. This is much higher than obtained by standard G-IBA treatment (62–64%), while it is in the upper range for advanced treatment on an industrial scale (70–85%; Allegrini et al., 2015; Lederer et al., 2025; Mehr et al., 2021). Since FB-IBA treatment has been rarely investigated, it was only possible to compare the extraction rate to that determined by Mühl et al. (2024) for metallic aluminum >4 mm, which was 98%. This is insignificantly higher than that determined in this study. In contrast, an extraction rate of 96% for metallic aluminum >4 mm was reported for G-IBA, which is higher than that observed in this study. This finding aligns with Vateva and Laner (2020), showing that a significant amount of metallic aluminum has a particle size <4 mm in G-IBA.

Figure 4 reveals that metallic aluminum is mainly extracted by ECS (88% and 78% extraction rate for FB-IBA and G-IBA, respectively). In FB-IBA, aluminum is additionally recovered by manual sorting, which implies that 8% of the aluminum particles have a particle size >50 mm. The ECS1 concentrate was obtained with a metallic aluminum content of 80% for both IBAs, while the ECS1 middlings fraction was obtained with 24% and 15% metallic aluminum content for FB- and G-IBA, respectively. Together, the ECS1 alone achieved 87% metallic aluminum extraction for FB-IBA and 73% for G-IBA processing. The second ECS (ECS2) plays a minor role in aluminum extraction, contributing 1% to the recovery from FB-IBA and 4% from G-IBA, while the content of metallic aluminum in the ECS2 product fraction is 6 wt-% in FB-IBA and 4 wt-% in G-IBA. During the treatment process, the material is screened at 8 mm between ECS1 and ECS2, and only material smaller than 8 mm passes through the ECS2. The oversize material is crushed and recirculated back into the input of the process (after sensor-based recovery of glass, in the case of FB-IBA). Crushing was reported to be advantageous for G-IBA treatment, as metallic particles are encapsulated in mineral agglomerates and are liberated upon comminution, thereby increasing the overall extraction efficiency (Šyc et al., 2020). Photos of ECS1 concentrate, ECS1 middlings, and ECS2 are shown in the Supplemental Section S3.1.1.

#### Grade- versus recovery-driven Eddy Current Separation

Eddy current separation is often described as a trade-off between recovery and grade (Rahman and Bakker, 2013). A recovery-driven operation minimizes the residual non-ferrous metal content in the mineral fraction but yields a low-quality product with lots of mineral impurities, while a grade-driven operation yields a high-quality product at the cost of insufficient recovery. The latter usually generates higher revenue, whereas a recovery-driven operation requires upgrading the metal concentrates to increase the revenue from the metals (Šyc et al., 2020). However, which exact configuration is chosen is highly company specific. The dry-wet treatment plant described in this study operates recovery-driven, combined with upgrading of the metal concentrates, since, to utilize the treated mineral fraction, a low residual metallic aluminum content in the treated mineral fraction is required.

#### Influence of the metallic particles’ degree of liberation

Another important parameter for metal extraction by eddy current separation is the degree of liberation of metallic particles, since mineral adhesions affect the particle trajectories and the content of mineral impurities in the product (Rahman and Bakker, 2013). It was demonstrated that high recovery and high grade can be achieved simultaneously in a single extraction step during dry treatment of dry-discharged G-IBA (Mehr et al., 2021). This is linked to the high degree of liberation of metallic particles in dry-discharge technology (Bunge, 2018). Therefore, also the high extraction rate observed for dry-discharged FB-IBA can be attributed to the complete liberated nature of metallic particles. However, recent studies have also shown that wet-discharged G-IBA can reach a similar degree of liberation if either electrodynamic fragmentation or intensive wet-sieving within 1 or 2 days after discharge is applied (Eggenberger, 2023b; Skutan, 2023b).

### Utilization of the treated mineral fraction

As already mentioned, the metallic aluminum content must be reduced as much as possible in the treated mineral fraction if it is intended to be used as IMAG in concrete (Shen et al., 2021). In addition to the target fractions of metallic aluminum (metal concentrates), the amount which is extracted into non-target fractions (glass and residues) also contributes to the overall extraction rate. However, this contribution is very limited, as itincreases the metal extraction rate by only 1% (Figure 4). Therefore, the overall extraction rate of metallic aluminum for the target and non-target fractions was determined to be 98% for the FB-IBA and 79% for G-IBA. Hence, 2% (FB-IBA) and 21% (G-IBA) of the initial metallic aluminum contained in the IBA remained in the mineral fraction after treatment. This corresponds to metallic aluminum contents of 0.5 wt-% (FB-IBA) and 1.0 wt-% (G-IBA).

Regarding the use as IMAG in concrete, the questions are whether the metallic aluminum contents are low enough so that they do not negatively influence the concrete strength, and what implications the encapsulated or liberated presence of metallic aluminum has. Although concrete production tests were not conducted, an estimate could be made based on what is known from scientific literature. In the Netherlands, a technical guideline for the use of IMAG produced from IBA limits the metallic aluminum content to 1.0 wt-% (Vateva et al., 2025). However, experimental findings with IMAG derived from Austrian IBAs revealed a divergent outcome. Aggregate from FB-IBA containing 0.2 wt-% and aggregate from G-IBA 0.9 wt-% metallic aluminum just met the minimum compressive strength limit for standard concrete (C25/30) with 12.5 wt-% substitution of natural aggregate (Lederer et al., 2025). Hence, these values can be interpreted as technical limit values. This shows that the metallic aluminum content must be lower in IMAG made from FB-IBA than in IMAG made from G-IBA to produce concrete with similar comprehensive strength. A possible explanation for this is that the aluminum particles occur liberated in FB-IBA and can therefore react with the surrounding alkalinity from the cement, while the mineral layers in wet-discharged G-IBA act as a barrier. This theory is underlined by the studies of Michalik et al. (2022) and Saffarzadeh et al. (2016), identifying a protective function of the quench minerals surrounding metallic aluminum particles against further corrosion by the quench water and also by a study from Switzerland on hard-to-recover residual metallic particles of wet-discharged G-IBA after dry treatment, which has found that up to 35–50% of the particle mass is mineral material (Skutan, 2023a). Photos of such particles are shown in the Supplemental Section S3.1.3.

From the perspective of metallic aluminum, the IMAG obtained from G-IBA after dry-wet treatment in this study might be applicable, depending on the substitution rate and concrete strength class. On the other hand, the limit value of 0.2 wt-% for IMAG obtained from FB-IBA is exceeded by 150%. It is likely that this impedes the use of the 0–8 mm mineral fraction as aggregate in any concrete strength classes due to the liberated, and hence reactive, metallic aluminum particles. Nonetheless, it is necessary to closely monitor the metallic aluminum content within the treated mineral fraction to determine if the obtained values in this study are representative of the current plant setup or represent peak values. Based on long-term monitoring of the process, it must be evaluated if further optimization and adjustments to the treatment process are required. An example for further optimization would be the separation of the mineral fraction <2 mm, which might be a viable method for producing IMAG with reliable low metallic aluminum content, as it was outlined in section “Comparison of the metallic aluminum content within industrial manufactured aggregate produced in other treatment plants.”

### Evaluation of the fluidized bed and grate incineration bottom ash used in this study

Some conclusions can be drawn from the observations on FB- and G-IBA in the dry-wet treatment made in this study. Advantages of FB-IBA include a low bed temperature and dry discharge, resulting in liberated metals without mineral adhesions and increased ECS trajectories. However, thin-walled items become very brittle when mechanically processed, leading to losses as metallic dust. The liberated state, on the other hand, is a disadvantage regarding the utilization of the mineral fraction as aggregate in concrete because metallic aluminum is highly reactive, and a very low residual content is required. In G-IBA, the wet-discharge leads to a mineral cover and the encapsulation of metallic particles, decreasing extraction efficiency; however, this mineral cover also acts as a barrier, lowering the reactivity of residual metallic aluminum when using the mineral fraction as IMAG in concrete. Since washing is applied in the investigated dry-wet process to utilize the mineral fraction, the material from both IBAs is wet when entering the eddy current separators. This reduces extraction efficiency compared to dry material (Rahman and Bakker, 2013) and necessitates a recovery-driven operation.

## Limitations

The study reports two large-scale treatment experiments using different IBA types in the same plant, mapping the facility’s material flows for metallic aluminum. After determining the metallic aluminum content in the process outputs, the overall content in the input (raw IBA) was reversely calculated. These values, however, represent only a snapshot, as IBA composition fluctuates over time. Reliable statements on metallic aluminum in raw IBA and on residual metal particles in the treated mineral fraction require long-term monitoring. The sample sizes in this study are also too small for general conclusions; Swiss experience indicates minimum sample masses of 1000 tonnes for raw IBA and 1 ton for treated mineral fractions to determine non-ferrous metal content (Eggenberger, 2023b).

The reported values refer only to the initial extraction of metallic aluminum from IBA. To assess the amount of aluminum which is recoverable and can be returned to the aluminum cycle, the upgrading of metal concentrates and the metal yield during remelting must also be considered. Swiss practice shows that at least 100 tonnes of extracted non-ferrous concentrate are needed to investigate the upgrading stage on an industrial scale (Eggenberger, 2023b). Aluminum losses during upgrading are highly process- and company-specific and cannot be generalized.

Determining metallic particles in IBA is challenging, and results vary strongly with the analytical method (Eggenberger, 2023a). This is critical where limit values apply to specific particle-size fractions – for example, non-ferrous metals >1 mm must be below 1.0% for landfilling in Switzerland (Swiss Federal Council, 2015), and >4 mm must be below 0.5% for use as IMAG in Austria (BMK, 2023). Mechanical processing can break metallic grains and shift them into smaller fractions, affecting results. For this reason, quantifying metallic aluminum in both coarse (>4 mm) and fine (<4 mm) fractions was essential to determine the total content. In G-IBA, pre-crushing with a vibrating roller may have fragmented coarse aluminum particles; in FB-IBA, brittle, flaky aluminum from dry discharge may fracture during sieving alone. Consequently, the distribution of aluminum above and below 4 mm carries unquantified uncertainty.

Using the soda-attack method for the <4 mm fraction is further limited by the small test portion size, a critical issue for highly heterogeneous materials (Ramsey et al., 2019). Although the samples were carefully prepared to minimize segregation effects, these cannot be eliminated. The method is also non-selective: metallic zinc is dissolved alongside aluminum, leading to overestimation where zinc is present, as in IBA (Vateva and Laner, 2020). This is particularly relevant for the treated mineral fraction, which is fully analyzed by the chemical method. Differentiating aluminum and zinc requires selective destruction and hand sorting combined with dense-media separation; applying this also to the <4 mm fraction would reduce non-specific soda-attack measurements and is recommended for future studies. If only the treated mineral fraction is assessed for aggregate use, determining the combined content of both metals is sufficient, as hydrogen gas evolution is the critical parameter (Chen et al., 2023).

## Conclusion

This study demonstrates that industrial-scale dry-wet treatment can achieve high extraction rates of metallic aluminum from both FB-IBA and G-IBA, although the two ash types behave differently due to their incineration conditions and resulting particle characteristics. FB-IBA enables almost complete aluminum extraction (98%) and yields a mineral fraction with low residual metal content. In contrast, wet-discharged G-IBA achieves 79% aluminum extraction, and the mineral fraction still contains a larger share of aluminum particles that persist in extraction despite advanced treatment. Since the obtained values represent only a snapshot of the continuous IBA waste stream, monitoring of the metallic aluminum content in the treated mineral fraction is required to evaluate if these values can be attributed to fluctuations in the IBA composition or if further optimization of the process is necessary to utilize the treated mineral fraction as IMAG in concrete. However, the findings highlight that the effectiveness of aluminum extraction is not determined by the treatment technology alone but is also influenced by upstream factors such as combustion temperature and discharge system, which determine the degree of metallic particle liberation in the IBA. These insights underline the need for IBA-type-specific treatment strategies, which require different considerations in the process design. Furthermore, how metal extraction from IBA is done in the industrial practice strongly depends on regional conditions and prevailing legislation, for example, if limit values for particulate metals for landfilling exist, what the requirements of scrap dealers or smelters for metal concentrates are, if specialized upgrading facilities are available, or what the limit values for the utilization of the treated mineral fraction as construction materials are. To quantify the ecological impact of the IBA management of both incineration technologies, experiments that include the entire value chain, from extraction to remelting of metals, must be conducted to obtain primary data for a life-cycle assessment.

## Supplemental Material

sj-pdf-1-wmr-10.1177_0734242X261444139 – Supplemental material for Extraction of metallic aluminum from fluidized bed and grate incineration bottom ash Supplemental material, sj-pdf-1-wmr-10.1177_0734242X261444139 for Extraction of metallic aluminum from fluidized bed and grate incineration bottom ash by Simon Mika, Julia Mühl and Jakob Lederer in Waste Management & Research
