# Supplementary material for: Extraction of metallic aluminum from fluidized bed and grate incineration bottom ash
Source: Waste Manag Res. 2026 May 2;44(9):1460–71. doi: 10.1177/0734242X261444139 (PMC13530342; doi:10.1177/0734242X261444139)
Supplement: Supplementary file 1 [file sj-pdf-1-wmr-10.1177_0734242X261444139.pdf]

Supplementary material: Extraction of metallic aluminium from fluidized bed and grate incineration bottom ash

Simon Mika, Julia Mühl, Jakob Lederer

Christian Doppler Laboratory for Design and Assessment of an efficient, recycling-based Circular Economy, Institute of Chemical, Environmental and Bioscience Engineering, TU Wien, Getreidemarkt 9/166, 1060 Vienna, Austria

1 Introduction

No supplementary material

2 Material and Methods

2.1 FB- and G-IBA origins

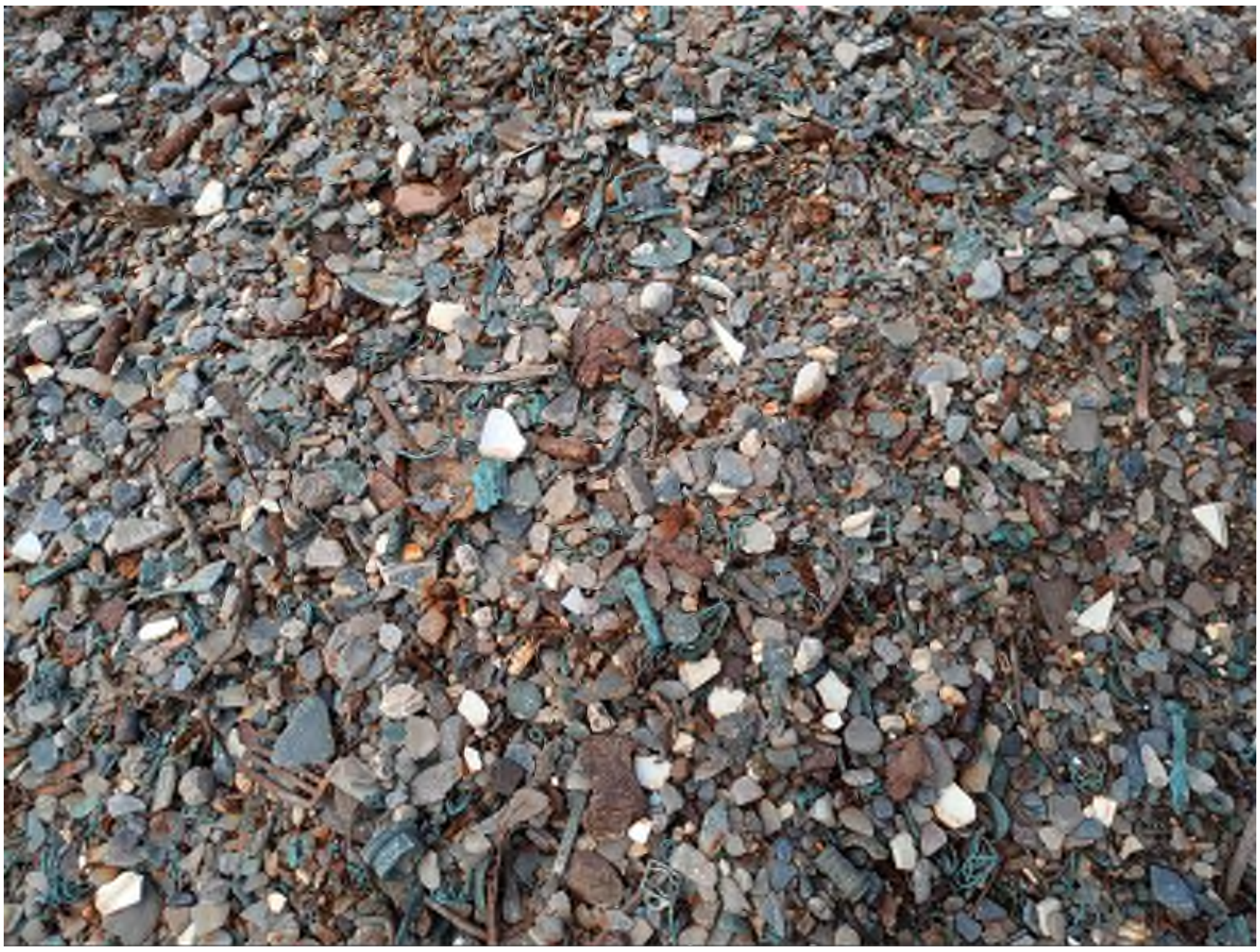

Figure 1: Untreated fluidized bed incineration bottom ash, dry-discharged, metals entirely liberated from minerals (picture taken by Julia Mühl with permission to be reproduced)

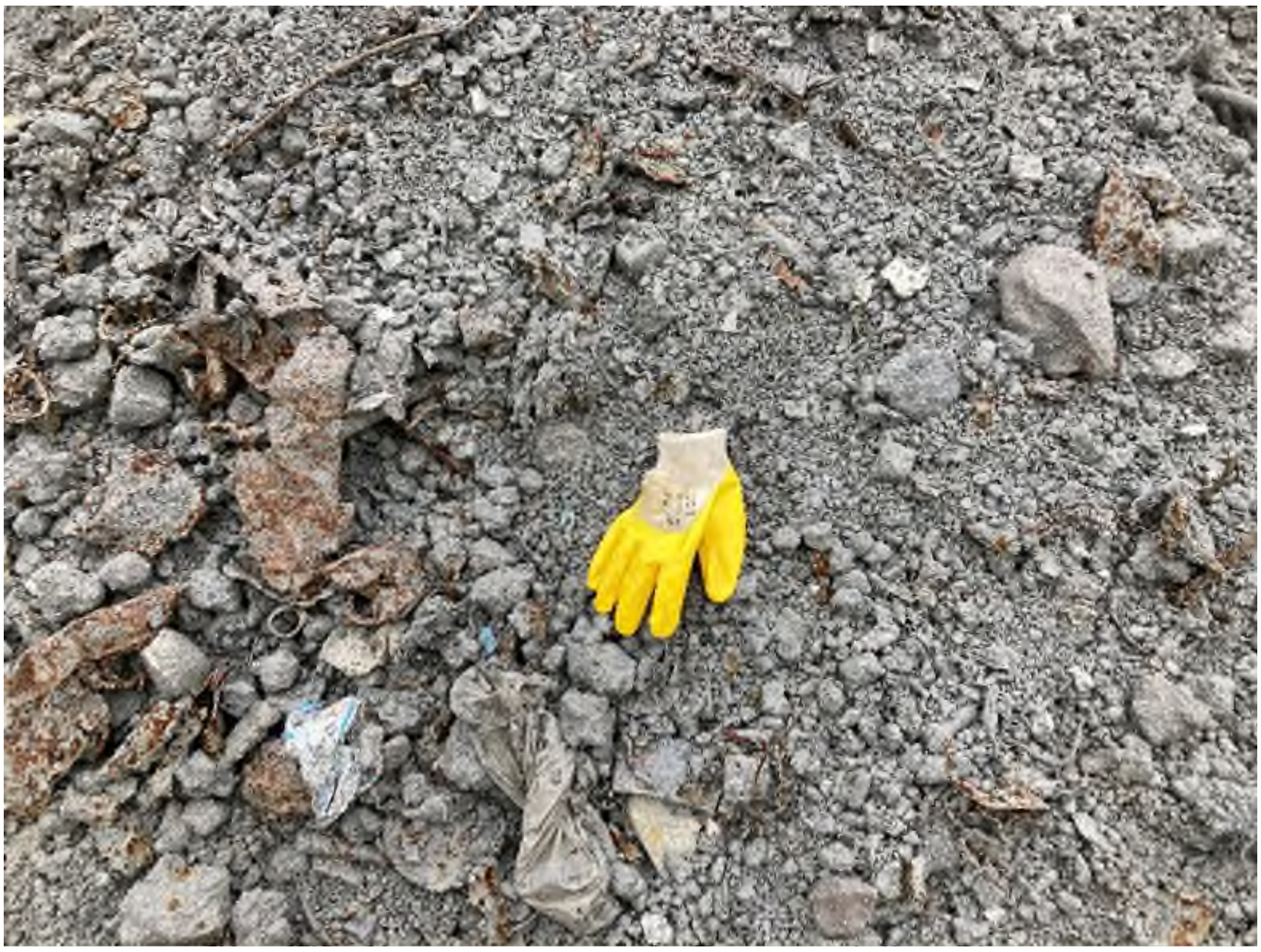

Figure 2: Untreated grate incineration bottom ash, wet-discharged, metals and minerals agglomerated (picture taken by Julia Mühl with permission to be reproduced)

2.2 Dry-Wet enhanced IBA treatment plant

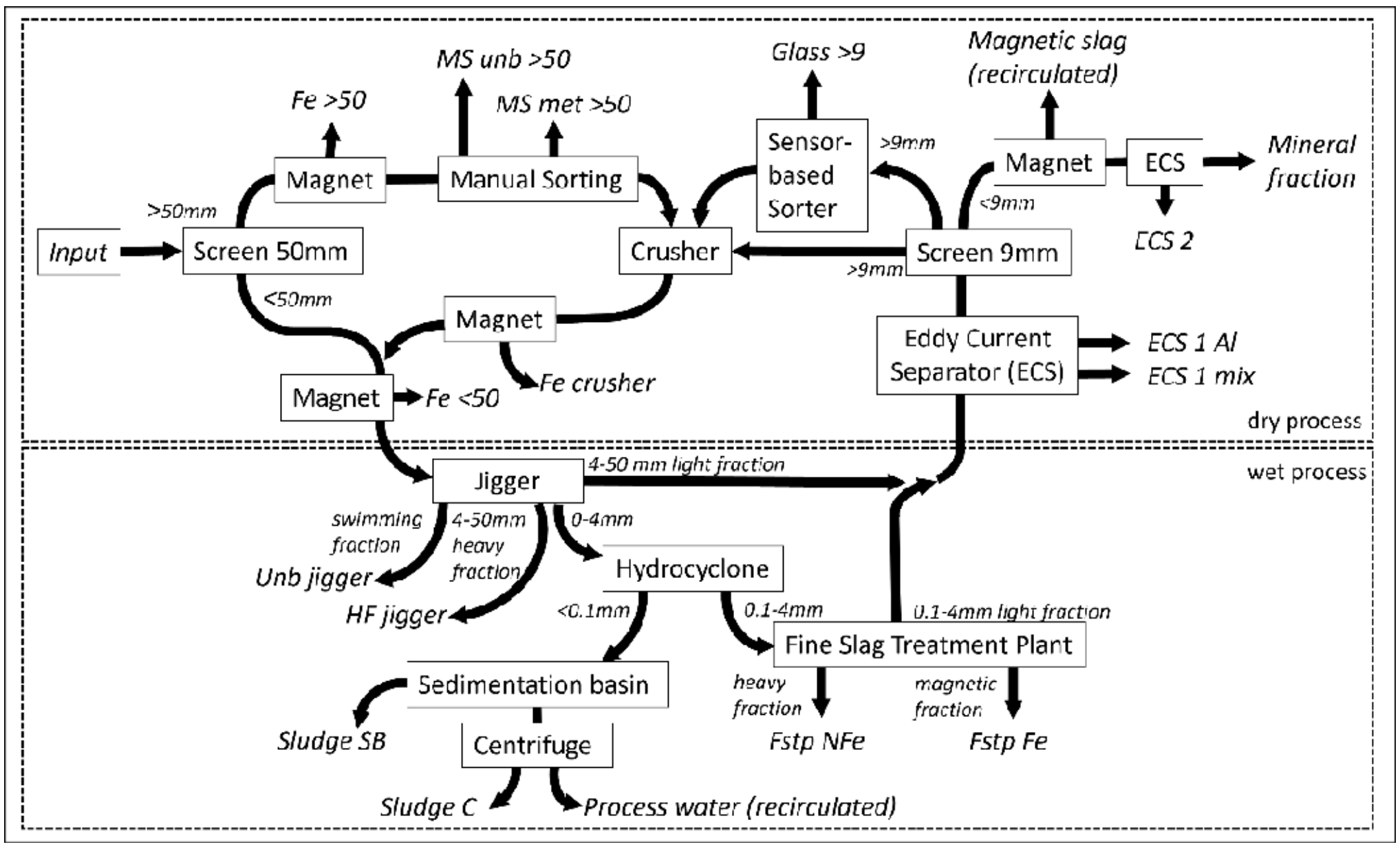

Figure 3: Process scheme of the dry-wet IBA treatment plant, reproduced from Mika et al. (2025) with permission

Table 1: Output flows of the enhanced dry-wet IBA treatment plant used for the experiments, from (Mühl et al., 2024).

Output flow j_{output}	Short name	Particle size range (mm)	Description of Output flow	Category
1	Fe >50	>50	Magnetic ferrous metals >50mm	Metal concentrates
2	MS met >50	>50	Manually sorted non-ferrous metals and stainless steel >50mm	Metal concentrates
3	MS unb >50	>50	Manually sorted unburnt material >50mm	Metal concentrates (contains missorted metals)
4	Fe crusher	0-50	Magnetic ferrous metals after crusher	Metal concentrates
5	Fe <50	0-50	Magnetic metals fraction separated prior to wet-jig	Metal concentrates
6	HF jigger	4-50	Wet-Jig output: heavy fraction (material with high density)	Metal concentrates
7	Unb jigger	4-50	Jigger output: floating material	Metal concentrates (contains e.g., aluminum foil)
8	Fstp Fe	0.1-4	Magnetic ferrous material fraction <4mm	Metal concentrates
9	Fstp NFe	0.1-4	Non-magnetic material fraction <4mm	Metal concentrates
10	Sludge SB	0-0.1	Sludge from sedimentation basin	Residues
11	Sludge C	0-0.1	Sludge from dewatering centrifuge	Residues
12	ECS 1 Al	4-50	Light non-ferrous metals from first ECS (concentrate fraction)	Metal concentrates
13	ECS 1 mix	4-50	Mixture of Aluminum, non-ferrous metals, stainless steel, minerals and glass from first ECS (middling fraction)	Metal concentrates
14	Glass >9	9-50	Glass fraction >9mm	Cullet
15	ECS 2	0.1-9	Non-magnetic metals from second ECS	Metal concentrates
16	MIN	0.1-9	Mineral fraction <9mm, can potentially be utilized as aggregate in concrete	Aggregate for concrete

2.3 IBA treatment experiments, sampling, and preparation of laboratory samples

The aspired sample size (or sample mass) was calculated in advance. The minimum sample mass depends on the particle size, the abundance and the distribution of the particles that carry a characteristic feature of interest (e.g., content of a substance i) and the desired precision for the determination of this characteristic feature (accepted sampling error) (Bunge & Bunge, 1999; Esbensen, 2019; Gy, 1998). Such particles are denoted as sub-goods in this study. An equation that considers all these attributes was proposed by Skutan and Brunner (2005) (Equation 1) and represents a modified version of the equation for minimum sampling mass by Bunge and Bunge (1999). It was used to calculate the minimum sampling mass with a relative standard deviation (rsd) of 10% for each output flow.

$$M_{i,j,p} = \frac{1}{\omega_{i,j,p}} \cdot \frac{\bar{c_i}^2}{{s_i}^2} \cdot \frac{1}{1 + 3\log\left(\sqrt[3]{\frac{m_{i,j,p\,max}}{m_{i,j,p\,10\%}}}\right)} \cdot m_{i,j,p\,max} \qquad (1)$$

In Equation 1, $M_{i,j,p}$ is the minimum sampling mass for the sub-good p carrying the substance i in an output flow j in kg and $\omega_{i,j,p}$ is the mass fraction (abundancy) of the sub-good p carrying the substance i in an output flow j in wt-%. In the presented case, the sub-goods p are metallic aluminum particles that are 100% made from the substance i, which is metallic aluminum (oxide layer and alloy elements were neglected).

The mean content of substance i is denoted as $\bar{c_i}$ in Equation 1 and given in mg/kg, while ${s_i}^2$ is the corresponding variance in mg²/kg². For the calculation, the following relation was considered: $\frac{\bar{c_i}^2}{{s_i}^2} = \frac{1}{rsd^2}$ .This allows to insert a target rsd value that can be arbitrarily selected (rsd=10% this study). When the mass distribution of an individual sub-good p that carries a specific substance i in an output flow j is considered, $m_{i,j,p\,max}$ is the maximum value in Equation 1 and $m_{i,j,p\,10\%}$ is the 10th percentile value of this distribution. These values were estimated from experience (Blasenbauer et al., 2023; Huber et al., 2020). However, the calculated sampling masses were only used to plan and carry out the sampling campaign, while the actual uncertainty values from the actually analyzed test sample masses were evaluated as part of the data analysis (see Section S2.4).

Table 2: Actually test portion sizes of the output flows for G- and FB-IBA, reproduced from Mika et al. (2025) with permission

Output flow	Test portion (kg)	Comment	Test portion (kg)	Comment
	G-IBA		FB-IBA	
Fe >50	-	not sampled, only content of electromagnetic coils estimated from scrap pile	26	Orientational sampling

MS met >50	152		608	85 kg bed material within spray cans included
MS unb >50	85		-	Not detected in FB-IBA
Fe crusher	-	not sampled, only content of electromagnetic coils estimated from scrap pile	22	Orientational sampling
Fe <50	75		9	
HF jigger metallic >4	31	Primary sample Hf jigger crushed with vibrating roller, sieved at 4 mm and both materials were split separately to test portions, unspecified: Test portion introduced to mill	26	Primary sample HF jigger was split to test portion; test portion was sieved at 4 mm and both materials were examined separately, unspecified: Test portion introduced to mill
HF jigger metallic <4 & non–metallic	11		5.5	
Unb jigger	1.5	Sample was sieved at 8 mm, >8 was only plastic film and not analysed, <8 = test portion analysed	6.9	sieved at 16mm, hand-sorting of >16 mm (mainly aluminium foil) and chemical analysis of <16 mm material
Sludge SB	3.9	Test portion introduced to mill	9.0	Test portion introduced to mill
Sludge C	2.0	Test portion introduced to mill	8.0	Test portion introduced to mill
Fstp Fe	4.8	Test portion introduced to mill	3.4	Test portion introduced to mill
Fstp NFe	4.6	Test portion introduced to mill	4.0	Test portion introduced to mill
ECS 1 Al metallic >4	11	ECS 1 Al test portion sieved at 4 mm and both materials were examined separately	13	ECS 1 Al test portion sieved at 4 mm and both materials were examined separately
ECS 1 Al metallic <4 & non–metallic	1.0		0.5	
ECS 1 mix metallic >4	10	Primary sample ECS 1 mix crushed with vibrating roller, sieved at 4 mm and both materials were split separately to test portions	13	Primary sample ECS 1 mix was split to test portion, test portion was sieved at 4 mm and both materials were examined separately
ECS1 mix metallic <4 & non–metallic	11		0.5	
Glass >9	-	not produced from G-IBA	120	Test portion introduced to crusher
ECS 2	1.9	Test portion introduced to mill	5.0	Test portion introduced to mill
MIN	55	Test portion introduced to crusher	60	Test portion introduced to crusher

Table 3: Sample preparation of output flows

Output flow j_{output}	Short name	Particle size range (mm)	Group of sample preparation and analysis	Preparation in G-IBA	Preparation in FB-IBA
1	Fe >50	>50	Group I	Handsorted,<br>Values from (Mühl et al., 2024)	Handsorted,<br>Values from (Mühl et al., 2024)
2	MS met >50	>50	Group I	Handsorted,<br>Values from (Mühl et al., 2024)	Handsorted,<br>Values from (Mühl et al., 2024)
3	MS unb >50	>50	Group I	Handsorted,<br>Values from (Mühl et al., 2024)	Handsorted,<br>Values from (Mühl et al., 2024)
4	Fe crusher	0-50	Group I	Handsorted,<br>Values from (Mühl et al., 2024)	Handsorted,<br>Values from (Mühl et al., 2024)
5	Fe <50	0-50	Group I	Handsorted,<br>Values from (Mühl et al., 2024)	Handsorted,<br>Values from (Mühl et al., 2024)

6	HF jigger	4-50	Group III	Spread on a metal plate and crushed with a vibrating roller to liberate coarse metals, sieving at 4mm, handsorting >4mm, fractionate milling <4mm	Due to nature of material immediate sieving at 4mm, handsorting >4mm, fractionate milling <4mm
7	Unb jigger	4-50	Group III	Sieving at 4mm, handsorting >4mm, fractionate milling <4mm	Sieving at 4mm, handsorting >4mm, fractionate milling <4mm
8	Fstp Fe	0.1-4	Group II	fractionate milling	fractionate milling
9	Fstp NFe	0.1-4	Group II	fractionate milling	fractionate milling
10	Sludge SB	0-0.1	Group II	total milling	total milling
11	Sludge C	0-0.1	Group II	total milling	total milling
12	ECS 1 Al	4-50	Group III	Crushed in a laboratory crusher to remove mineral adheasions from quenching, Sieving at 4mm, handsorting >4mm, fractionate milling <4mm	Due to nature of material immediate sieving at 4mm, handsorting >4mm, fractionate milling <4mm
13	ECS 1 mix	4-50	Group III	Spread on a metal plate and crushed with a vibrating roller to liberate coarse metals, sieving at 4mm, handsorting >4mm, fractionate milling <4mm	Due to nature of material immediate sieving at 4mm, handsorting >4mm, fractionate milling <4mm
14	Glass >9	9-50	Group III	Not recovered	Handsorting of metals >4mm, crushing in a laboratory crusher, sieving at 1mm and handsorting of metals >1mm, total milling of <1mm
15	ECS 2	0-8	Group II	fractionate milling	fractionate milling
16	MIN	0-8	Group II	fractionate milling	fractionate milling

2.4 Determination of metallic aluminum contents

2.4.1 Determination of metallic aluminum by the soda-attack method

The method is based on the volumetric measurement of hydrogen gas, which evolves at the reaction of metallic aluminum with concentrated sodium hydroxide solution. The chemical reaction is:

$$2Al^0 + 2NaOH + 6H_2O = 2Na[Al(OH)_4] + 3H_2$$

By application of the ideal-gas equation, the mol of hydrogen are calculated based on the volume produced.

$$p \cdot V = n \cdot R \cdot T$$

Therefore, it was necessary to measure the temperature and the air pressure in the laboratory. The apparatus used is shown in Figure 4.

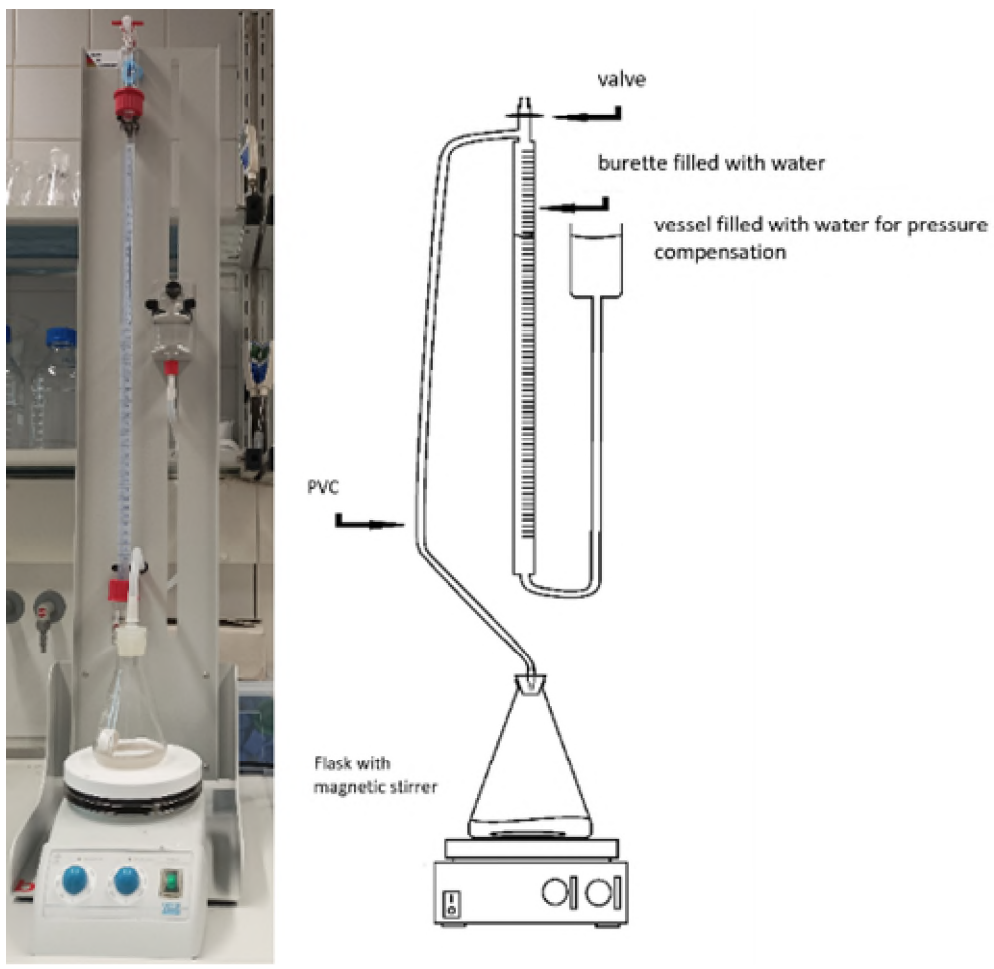

Figure 4: Apparatus used for the determination of metallic aluminum by soda-attack method (behrotest® Calcimeter SCM 1)

Milled samples (<0.1mm or 0.1-0.5mm) were used for the experiments and triplicate measurements were performed. Before the sample was measured, the apparatus was tested for its functionality by performing experiments with household aluminum foil. For measurements, 5-10g samples were weighed into the Erlenmeyer flask, and a small container with 30% NaOH solution was inserted into the flask. After closing the apparatus, the magnetic stirring was turned on, which flipped the small container, starting the reaction between metallic aluminum (Al^0) and NaOH.

2.4.2 Determination of the variance based on sample size and heterogeneity of the material

The unknown variable is the variance (s^2), since sSD can be obtained by taking the square root. From a general consideration, the following relation can be drawn: while the substance content c is a constant value, there are two parts contributing to the sSD of the content c. One part originates in the uncertainty of the substance content c itself, due to random errors e.g., measurement uncertainties. This part will be denoted as $RSD_{measurement}$. The second part originates in the heterogeneity of the sampled material flow and the analyzed sample size/mass. This part will be denoted as $RSD_{sample\ mass}$ in the following section. Equation 2 shows this relationship.

$$s^2 = c^2 \cdot \left(RSD_{measurement}^2 + RSD_{sample\ mass}^2\right) \quad (2)$$

where s^2 is the variance of the content of substance i in mg²/kg², c is the content of substance i in the sample in mg/kg, $RSD_{measurement}$ is the RSD of measured substance content i and $RSD_{sample\ mass}$ is the RSD of the substance content i deriving from the analyzed sample mass.

This Equation is the basis for the calculation of any sSD values. As mentioned in Section 2.3 in the manuscript, three different approaches were used to characterize the output flows.

For those that were only characterized by hand-sorting, $RSD^2_{measurement}$ was set to zero, while $RSD^2_{sample\ mass}$ was calculated according to Equation 3, which was rearranged from Equation 1, considering: $\frac{\bar{c}_i{}^2}{s_i{}^2} = \frac{1}{rsd^2}$:

$$RSD_{i,j,p,sample\ mass} = \sqrt{\left(\frac{1}{\omega_{i,j,p}} \cdot \frac{1}{M_{i;j;p}} \cdot \frac{1}{1+3\log\left(\sqrt[3]{\frac{m_{j,p,max}}{m_{j,p,10\%}}}\right)} \cdot m_{j,p,\max}\right)} \qquad (3)$$

For those samples that were totally or fractionally milled and chemically analyzed, Equation 1 could not be applied because no information on the abundance of the individual particles was available. In this cases, Equation 4 was used to calculate $RSD^2_{sample\ mass}$, which is a simplification of “Gy’s formula” for the “fundamental sampling error” and is usually applied for environmental or geological samples. Tabulated values were used for the constant factors as suggested by Gerlach and Nocerino (2003).

$$RSD_{i,j,sample\ mass} = \sqrt{\left(\frac{\rho \cdot l \cdot f \cdot g \cdot d^3}{c_{i,j} \cdot M_j}\right)} \qquad (4)$$

where $RSD_{i,j,sample\ mass}$ is the RSD of the content of substance i in the sub-good p deriving from the test portion mass, ρ is the density of the sub-good p (ρ = specific densities for native metals, 2.5 g/cm³ for mineral and glass particles, $c_{i,j}$ the content of substance i in the output flow j_2, l the liberation factor (l = 1 for environmental applications (max. heterogeneity), = 0.2 for glass), f the particle shape factor (f = 0.5 for minerals and metals, = 0.1 for glass), g the particle size range factor (g = 0.4 for material passing through a screen), d^3 the nominal size of the particles (in cm³) and M_j is the mass of the test portion introduced to the first comminution step.

$RSD_{Al,measurement}$ was obtained from duplicate measurements with the soda-attack method. Samples that were milled in fractions, which were analyzed separately, the variance of each fraction was weighted according to their mass share and then the sum variance of all fractions was formed.

3 Results and Discussion

3.1 Metallic aluminum determined in the treatment units of the IBA treatment plant

Table 4: *Material flows (t, DM/8h), determined metallic aluminum content (wt-%) and calculated material flows of metallic aluminum kg, DMt/8h) and content of metallic aluminum per t IBA, DM of **FB-IBA treatment experiments***

Output flow short name	Output material flows [t, DM/8h]	Content of metallic aluminum [wt-%]	Material flow of metallic aluminum [kg, DM/8h]	Content of metallic aluminum [kg/t IBA, DM]	Determination of met.Al. Handsorting/ Chemical Analysis	Category
Fe >50	5.5	0.7	39	0.21	100% / 0%	Metal concentrates
MS met >50	4.6	22	1000	5.5	100% / 0%	Metal concentrates
MS unb >50	-	-	-	-	100% / 0%	Metal concentrates (contains missorted metals)
Fe crusher	3.4	1.0	34	0.18	100% / 0%	Metal concentrates
Fe <50	3.7	0.5	18	0.10	100% / 0%	Metal concentrates
HF jigger	2.9	0.8	23	0.12	83% / 17%	Metal concentrates
Unb jigger	0.11	17	19	0.10	17% / 83%	Metal concentrates (contains e.g., aluminum foil)
Fstp Fe	0.37	0.0	0	0.00	0% / 100%	Metal concentrates
Fstp NFe	0.08	0.0	0	0.00	0% / 100%	Metal concentrates
Sludge SB	8.2	0.4	33	0.18	0% / 100%	Residues
Sludge C	1.3	0.2	2.5	0.01	0% / 100%	Residues
ECS 1 Al	9.5	80	7600	40	97% / 3%	Metal concentrates
ECS 1 Mix	17	23	3900	21	96% / 4%	Metal concentrates
Glass >9	65	0.1	0	0.35	1% / 99%	Cullet
ECS 2	2.1	6.4	130	0.7	0% / 100%	Metal concentrates
MIN	63	0.5	310	1.7	0% / 100%	Aggregate for concrete

Table 5: Material flows (t, DM/8h), determined metallic aluminum content (wt-%) and calculated material flows of metallic aluminum kg, DMt/8h) and content of metallic aluminum per t IBA, DM of ***G-IBA treatment experiments***

Output flow short name	Output material flows [t, DM/8h]	Content of metallic aluminum [wt-%]	Material flow of metallic aluminum [kg, DM/8h]	Content of metallic aluminum [kg/t IBA, DM]	Determination of met.Al. Handsorting/ Chemical Analysis	Category
Fe >50	1.3	0.0	0	0.00	100% / 0%	Metal concentrates
MS met >50	1.7	3.2	54	0.28	100% / 0%	Metal concentrates
MS unb >50	0.3	0.0	0	0.00	100% / 0%	Metal concentrates (contains misssorted metals)
Fe crusher	0.25	0.0	0	0.00	100% / 0%	Metal concentrates
Fe <50	4.7	0.0	0	0.00	100% / 0%	Metal concentrates
HF jigger	8.0	0.9	72	0.38	40% / 60%	Metal concentrates
Unb jigger	0.59	0.6	3.5	0.02	0% / 100%	Metal concentrates
Fstp Fe	1.9	0.0	0	0.00	0% / 100%	Metal concentrates
Fstp NFe	0.36	0.0	0	0.00	0% / 100%	Metal concentrates
Sludge SB	15	0.1	15	0.08	0% / 100%	Residues
Sludge C	5.5	0.1	5.5	0.03	0% / 100%	Residues
ECS 1 Al	3.2	80	2600	13	92% / 8%	Metal concentrates
ECS 1 Mix	14	15	2100	11	57% / 43%	Metal concentrates
Glass >9	-	-	-	-	-	Cullet
ECS 2	5.3	4.2	220	1.2	0% / 100%	Metal concentrates
MIN	130	1.0	1300	6.80	0% / 100%	Aggregates for concrete

Table 6: Calculation of relative standard deviation for FB-IBA output flows including error from sampling mass and measurement

**FB-IBA**			$RSD_{i,j,>4\,mm} = \sqrt{\left(\frac{1}{\omega_{i,j,p}} \cdot \frac{1}{M_j} \cdot \frac{1}{1+3\log\left(\sqrt[3]{\frac{m_{j,p,max}}{m_{j,p,10\%}}}\right)} \cdot m_{j,p,\max}\right)}$					$RSD_{i,j\,<4mm} = RSD_{i,j,<4mm,sample\,mass} + RSD_{i,j,<4\,mm,measurement}$									$RSD_{Al,j} = RSD_{>8mm} \cdot \omega_{>8mm} + RSD_{4-8mm} \cdot \omega_{4-8mm} + RSD_{<4mm} \cdot \omega_{<4mm}$
	Particle size of test sample (mm)	Mass fraction	M_j (g)	ω_{Al} (kg/kg)	$m_{Al,max}$ (g) *	$m_{Al,10\%}$ (g) *	$RSD_{Al,>4\,mm}$ (-)	M_j (g)	c_{Al} (kg/kg)	ρ_{Al} (g/cm³)	f(-) ˣ	g(-) ˣ	l(-) ˣ	d_{max} (cm)	$RSD_{Al,sample\,mass} = \sqrt{\left(\frac{\rho \cdot l \cdot f \cdot g \cdot d^3}{c_{i,j} \cdot M_j}\right)}$	$RSD_{Al,measurement}$	
Fe >50	>50	-	26 444	0.007	25	5	1%	-	-	-	-	-	-	-	-	-	1%
MS met >50	>50		607 793	0.22	100	10	2%	-	-	-	-	-	-	-	-	-	2%
MS unb >50	>50	-	-	-	-	-	-	-	-	-	-	-	-	-	-	-	-
Fe crusher	<50	-	22 300	0.006	25	5	5%	-	-	-	-	-	-	-	-	-	5%
Fe <50	<50	-	9 391	0.003	25	5	45%	-	-	-	-	-	-	-	-	-	45%
HF jigger	>8	56%	18 425	0.013	100	10	47%	-	-	-	-	-	-	-	-	-	
	4-8	27%	8 633	0.002	10	1	59%	-	-	-	-	-	-	-	-	-	43%
	<4	17%	-	-	-	-	-	5 470	0.002	2.7	0.5	0.4	1	0.4	6%	2%	
Unb jigger	>16	17%	3 563	0.92	5	1	3%	-	-	-	-	-	-	-	-	-	2%
	<16	83%		-	-	-	-	6 565	0.02	2.7	0.5	0.4	1	1.6	13%	2%	
Fstp Fe	<4	-	-	-	-	-	-	3 400	0.0002	2.7	0.5	0.4	1	0.4	22%	21%	31%
Fstp NFe	<4	-	-	-	-	-	-	4 000	0.0002	2.7	0.5	0.4	1	0.4	21%	31%	38%
Sludge SB	<0.1	-	-	-	-	-	-	9 000	0.0042	2.7	0.5	0.4	1	0.01	0.01%	0.2%	0.2%
Sludge C	<0.1	-	-	-	-	-	-	8 000	0.0017	2.7	0.5	0.4	1	0.01	0.02%	1.4%	1.4%
ECS 1 Al	>4	97%	13 417		50	5	15%	-	-	-	-	-	-	-	-	-	14%
	<4	3%	-	-	-	-	-	414	0.035	2.7	0.5	0.4	1	0.4	5%	2.5%	
ECS 1 Mix	>4	96%	12 978	0.2407	30	1	15%	-	-	-	-	-	-	-	-	-	14%
	<4	4%	-	-	-	-	-	479	0.0854	2.7	0.5	0.4	1	0.4	3%	0.8%	
Glass >9	>4	1%	1 332	0.003	10	1	10%	-	-	-	-	-	-	-	-	-	6%
	<4	99%	-	-	-	-	-	117 351	0.0007	2.7	0.5	0.4	1	0.4	0.4%	6%	
ECS 2	<8	-	-	-	-	-	-	5 000	0.0635	2.7	0.5	0.4	1	0.8	5%	0.1%	5%
MIN	<8	-	-	-	-	-	-	60 000	0.0049	2.7	0.5	0.4	1	0.8	4%	0.2%	4%

*Estimated

ˣ from (Gerlach & Nocerino, 2003): l the liberation factor (l = 1 for environmental applications (max. heterogeneity), = 0.2 for glass), f the particle shape factor (f = 0.5 for minerals and metals, f = 0.1 for glass), g the particle size range factor (g = 0.4 for material passing through a screen)

Table 7: Calculation of relative standard deviation for G-IBA output flows including error from sampling mass and measurement

**G-IBA**			$RSD_{i,j,>4\,mm} = \sqrt{\left(\frac{1}{\omega_{i,j,p}} \cdot \frac{1}{M_j} \cdot \frac{1}{1+3\log\left(\sqrt[3]{\frac{m_{j,p,max}}{m_{j,p,10\%}}}\right)} \cdot m_{j,p,\max}\right)}$					$RSD_{i,j\,<4mm} = RSD_{i,j,<4mm,sample\ mass} + RSD_{i,j,<4\,mm,measurement}$									$RSD_{Al,j} = RSD_{>8mm} \cdot \omega_{>8mm} + RSD_{4-8mm} \cdot \omega_{4-8mm} + RSD_{<4mm} \cdot \omega_{<4mm}$
	Particle size of test sample (mm)	Mass fraction	M_j (g)	ω_{Al} (kg/kg)	$m_{Al,max}$ (g) *	$m_{Al,10\%}$(g) *	$RSD_{Al,>4\,mm}$ (-)	M_j (g)	c_{Al} (kg/kg)	ρ_{Al} (g/cm³)	f(-) ˣ	g(-) ˣ	l(-) ˣ	dmax (cm)	$RSD_{Al,sample\ mass} = \sqrt{\left(\frac{\rho \cdot l \cdot f \cdot g \cdot d^3}{c_{i,j} \cdot M_j}\right)}$	$RSD_{Al,measurement}$	
Fe >50	-	-	-	-	-	-	-	-	-	-	-	-	-	-	-	-	-
MS met >50	>50	-	151 910	0.03	150	10	10%	-	-	-	-	-	-	-	-	-	10%
MS unb >50	>50	-	84 500	0.04	100	10	34%	-	-	-	-	-	-	-	-	-	34%
Fe crusher	-	-	-	-	-	-	-	-	-	-	-	-	-	-	-	-	-
Fe <50	-	-	-	-	-	-	-	-	-	-	-	-	-	-	-	-	-
HF jigger	>8	16%	22 314	0.005	50	5	49%	-	-	-	-	-	-	-	-	-	31%
	4-8	25%	8 774	0.025	10	2	16%	-	-	-	-	-	-	-	-	-	
	<4	60%	-	-	-	-	-	10 605	0.0017	2.7	0.5	0.4	1		4%	4%	
Unb jigger	<50	-	-	-	-	-	-	1 491	0.00008	2.7	0.5	0.4	1	0.8	149%	7%	149%
Fstp Fe	<4	-	-	-	-	-	-	4 759	0.000028	2.7	0.5	0.4	1	0.4	16%	12%	20%
Fstp NFe	<4	-	-	-	-	-	-	4 628	0.00001	2.7	0.5	0.4	1	0.4	74%	2%	74%
Sludge SB	<0.1	-	-	-	-	-	-	3 866	0.0013	2.7	0.5	0.4	1	0.01	0.032%	0.2%	0.2%
Sludge C	<0.1	-	-	-	-	-	-	2 000	0.0014	2.7	0.5	0.4	1		0.044%	5%	2%
ECS 1 Al	>4	92%	10 497	0.81	100	5	7%	-	-	-	-	-	-	-	-	-	15%
	<4	8%	-	-	-	-	-	958	0.1700	2.7	0.5	0.4	1	0.4	2%	10%	
ECS 1 Mix	>8	9%	8 748	0.58	100	10	10%	-	-	-	-	-	-	-	-	-	28%
	4-8	48%	768	0.29	25	5	26%	-	-	-	-	-	-	-	-	-	
	<4	43%	-	-	-	-	-	10 987	0.0210	2.7	0.5	0.4	1	0.4	1%	9.4%	
Glass >9	-	-	-	-	-	-	-	-	-	-	-	-	-	-	-	-	-
ECS 2	<8	-	-	-	-	-	-	1 886	0.03057	2.7	0.5	0.4	1	0.4	10%	7%	29%
MIN	<8	-	-	-	-	-	-	55 400	0.061	2.7	0.5	0.4	1	0.8	1%	2%	2%

*Estimated

ˣ from (Gerlach & Nocerino, 2003): l the liberation factor (l = 1 for environmental applications (max. heterogeneity), = 0.2 for glass), f the particle shape factor (f = 0.5 for minerals and metals, f = 0.1 for glass), g the particle size range factor (g = 0.4 for material passing through a screen)

3.1.1 Target fractions for metallic aluminium (metal concentrates)

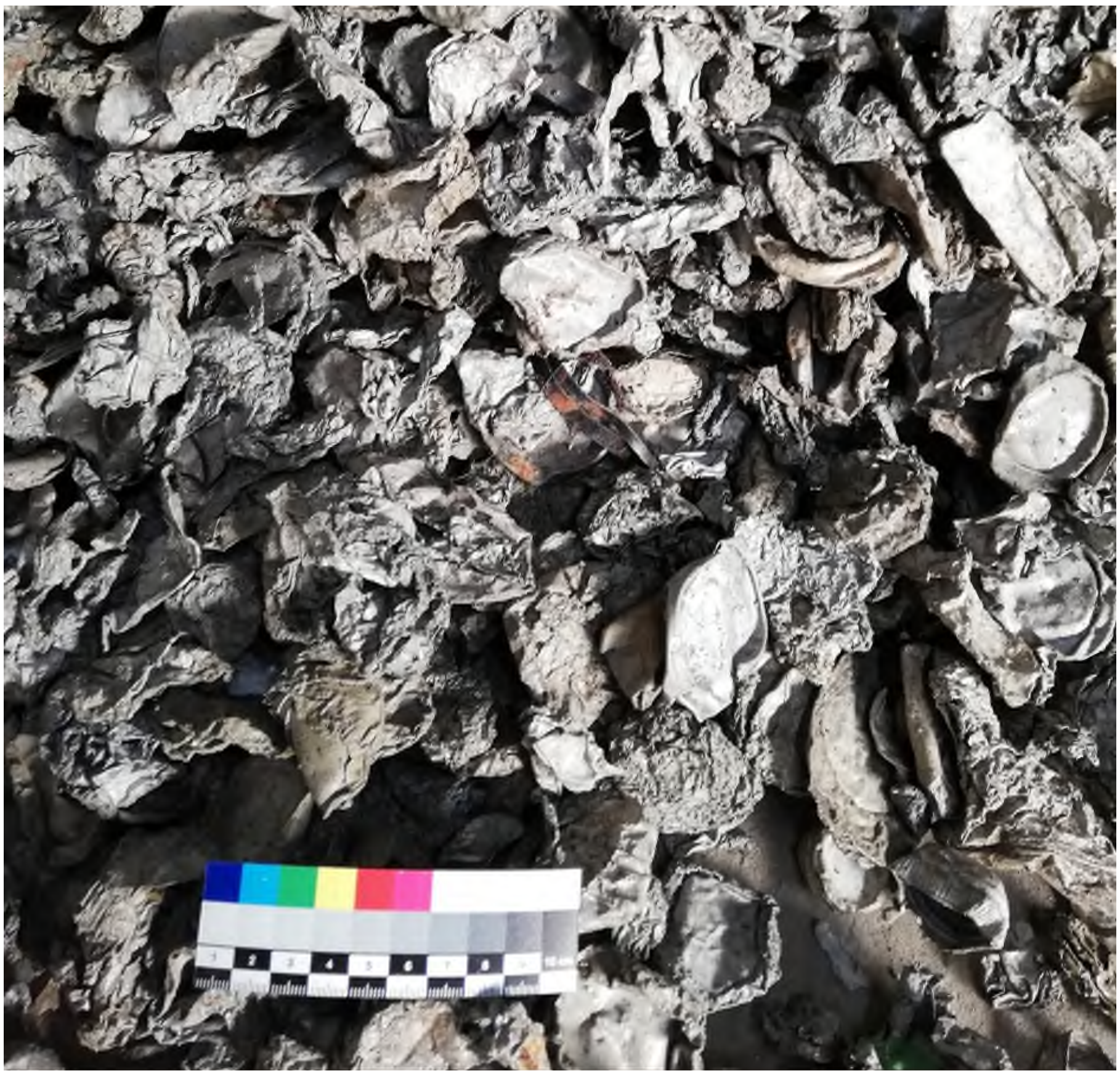

Figure 5: ECS1 concentrate from FB-IBA (picture taken by Julia Mühl with permission to be used)

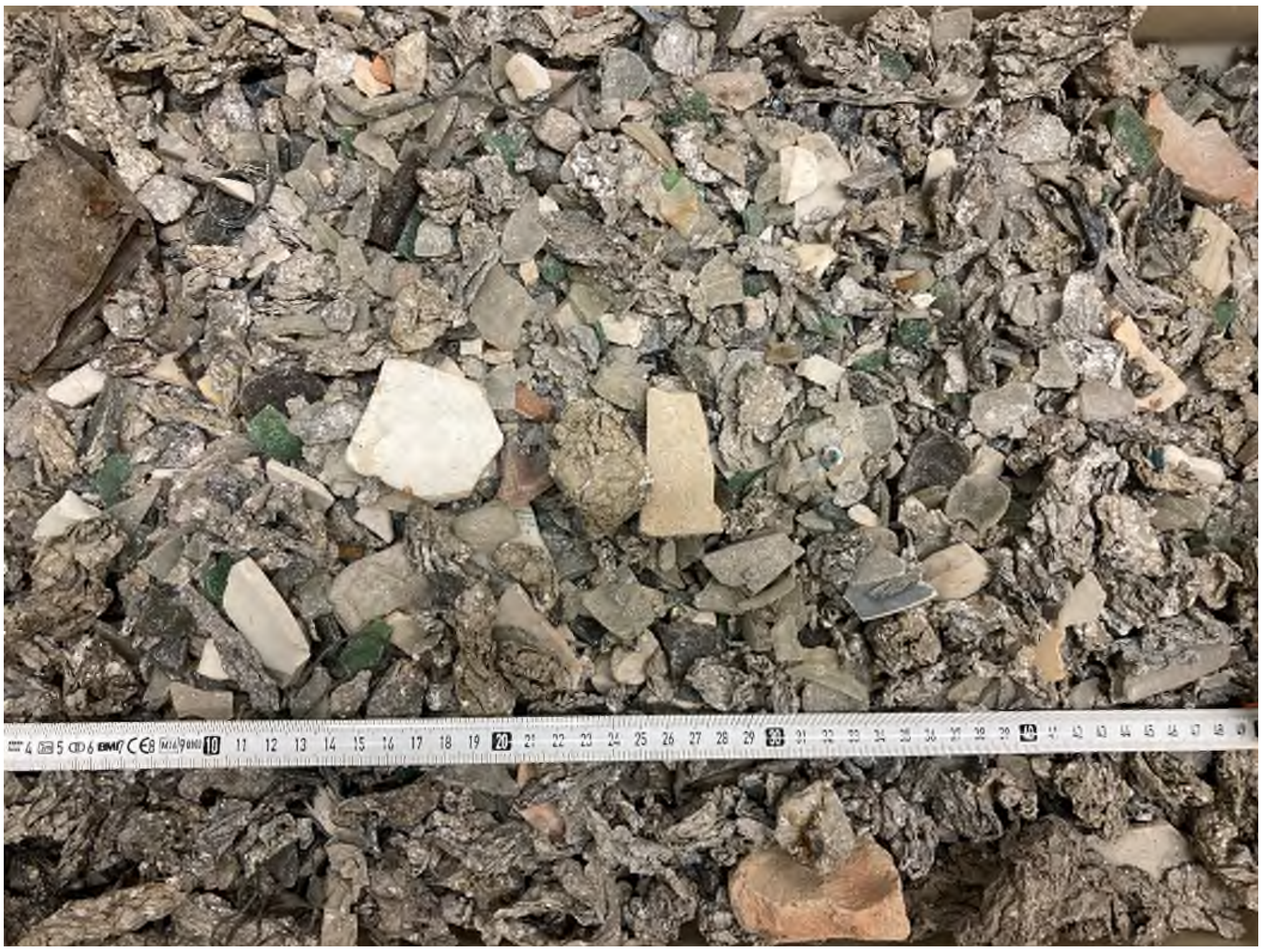

Figure 6: ECS1 middlings from FB-IBA (own picture)

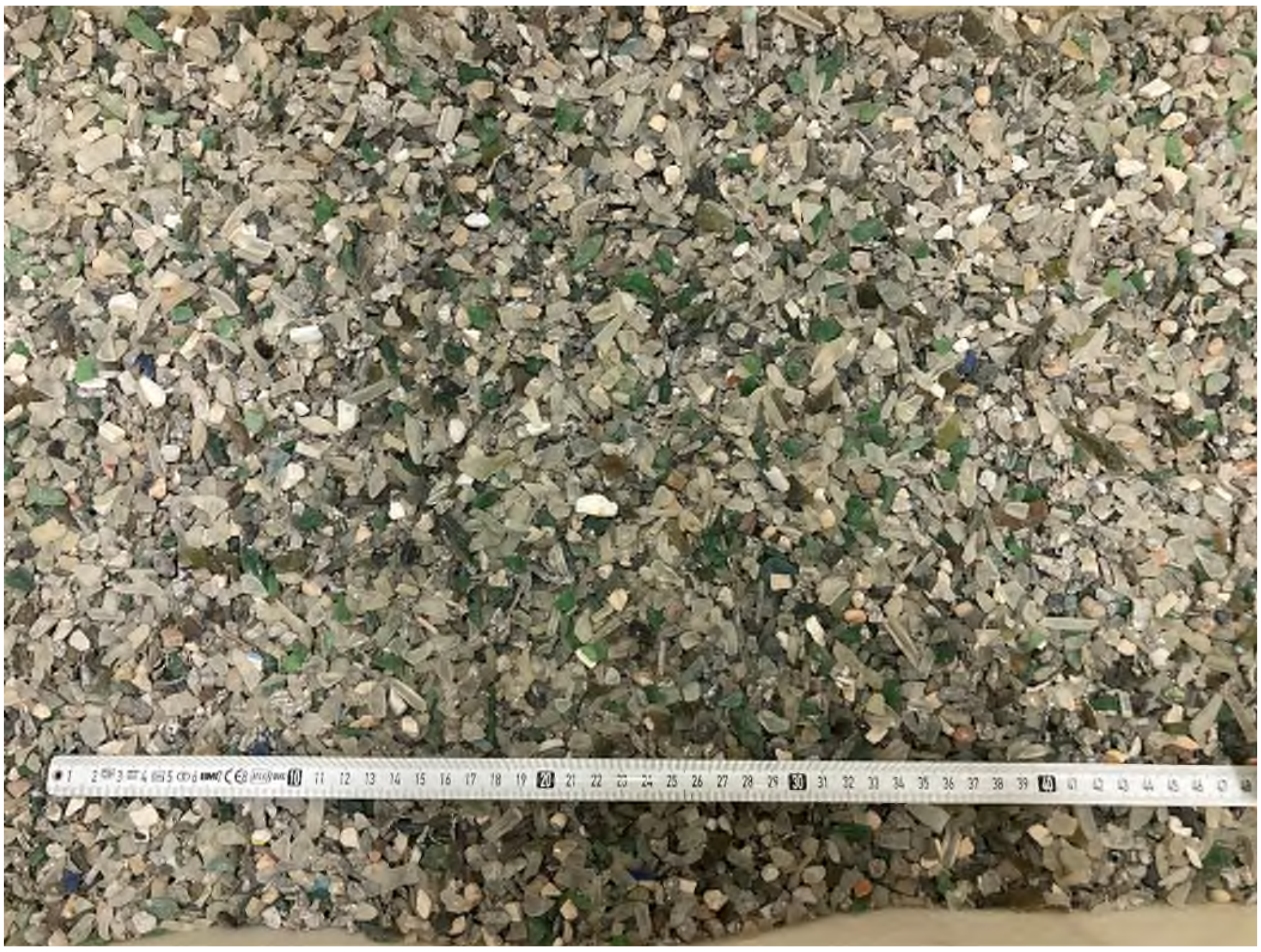

Figure 7: ESC2 non-ferrous metal product from FB-IBA (own picture)

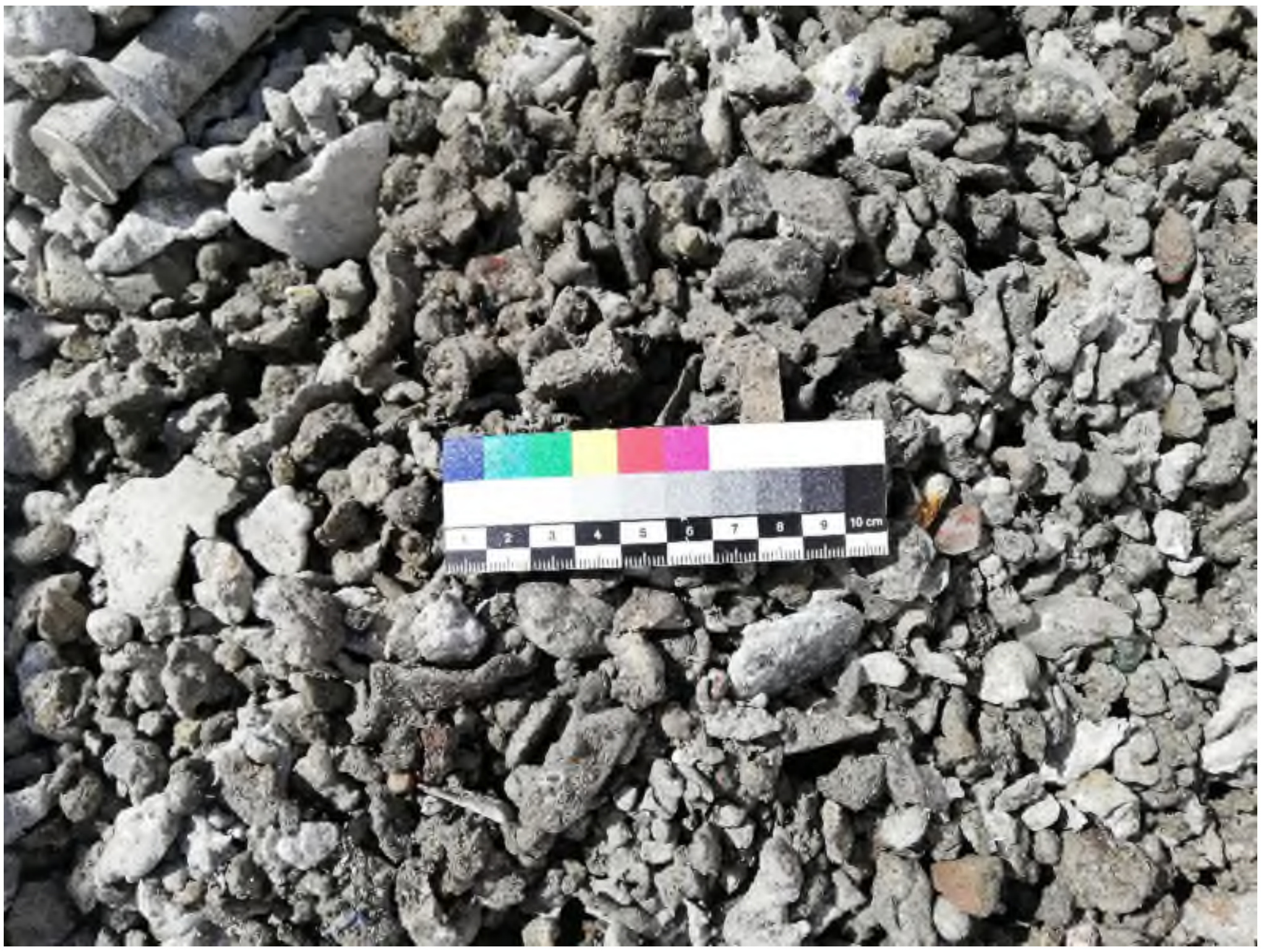

Figure 8: ECS1 concentrate from G-IBA (picture taken by Julia Mühl with permission to be used)

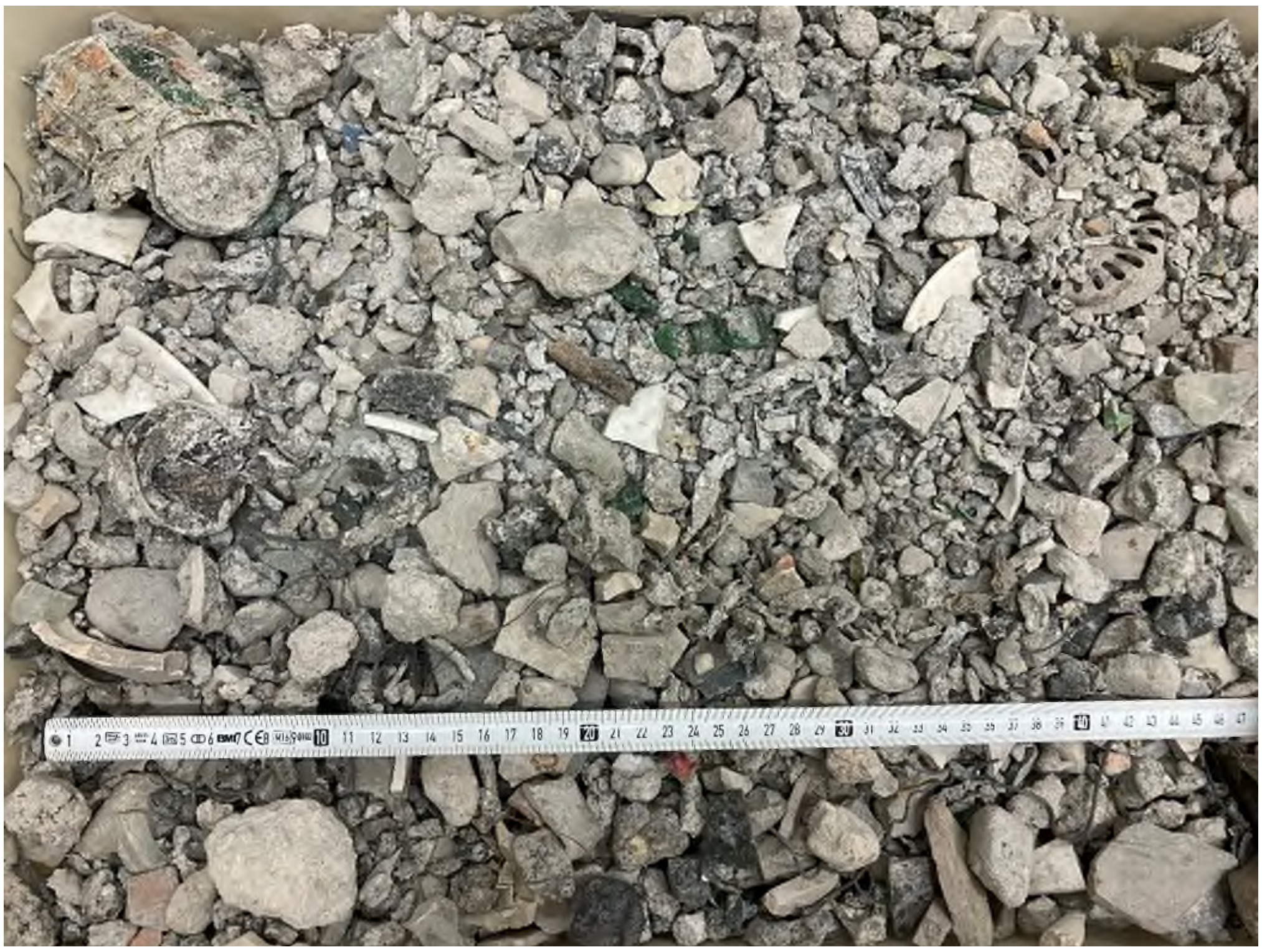

Figure 9: ECS1 middling from G-IBA (own picture)

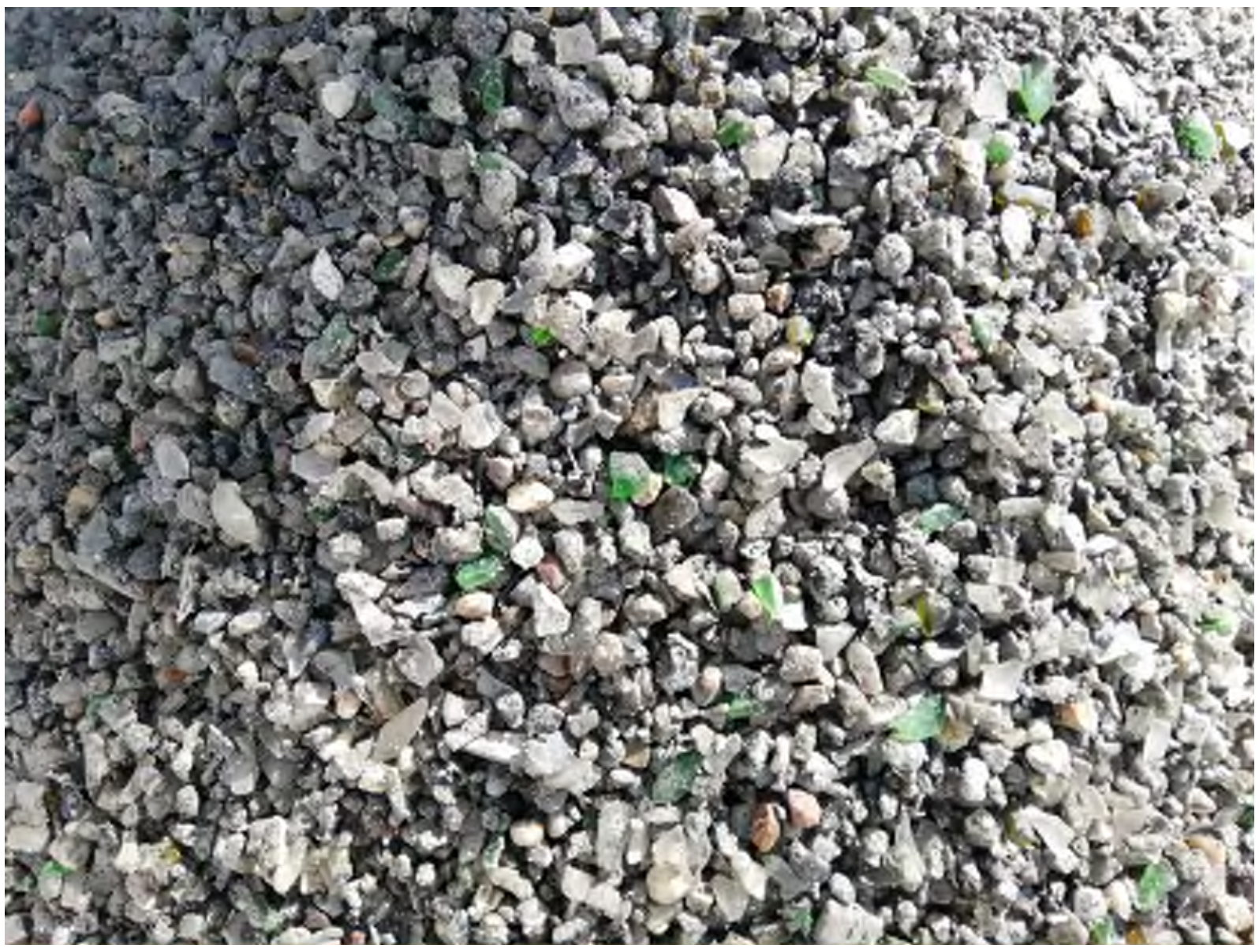

Figure 10: ECS2 concentrate from G-IBA (picture taken by Julia Mühl with permission to be used)

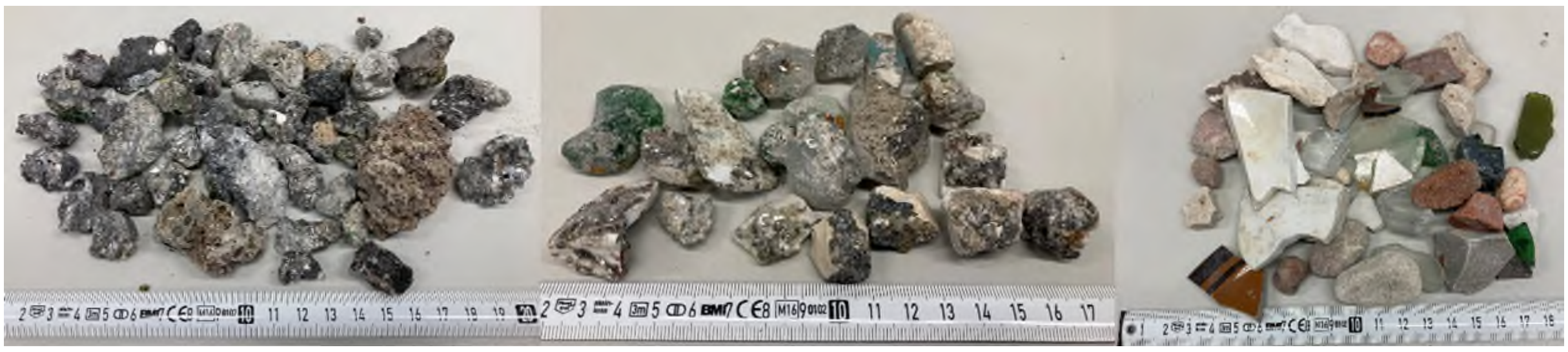

Figure 11: Agglomerates (left), Refractory minerals with quench-mineral layer (middle), Refractory minerals (right) from G-IBA (own pictures)

3.1.2 Non-Target fractions for metallic aluminium (Glass and Residues)

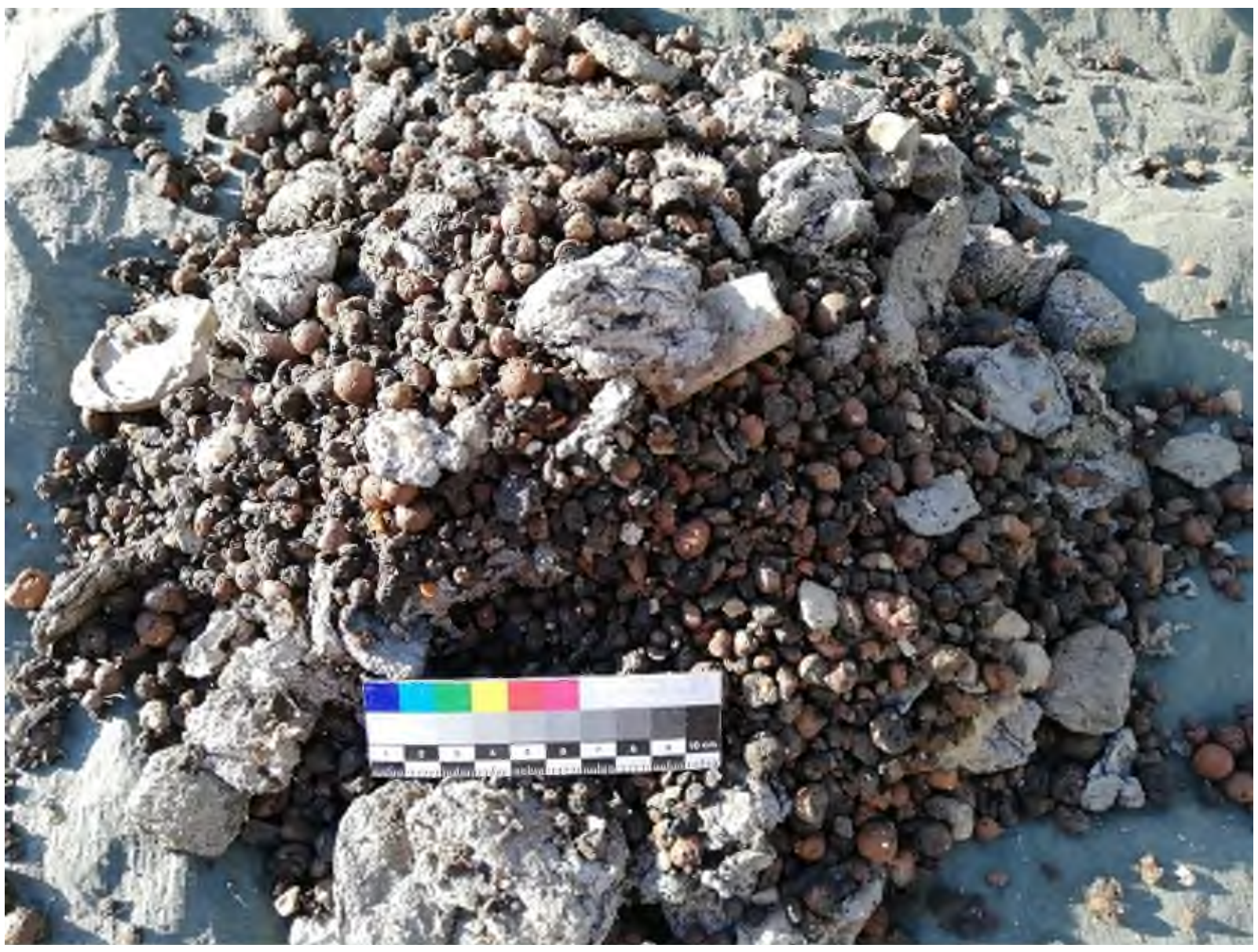

Figure 12: Swimming material from FB-IBA (picture taken by Julia Mühl with permission to be used)

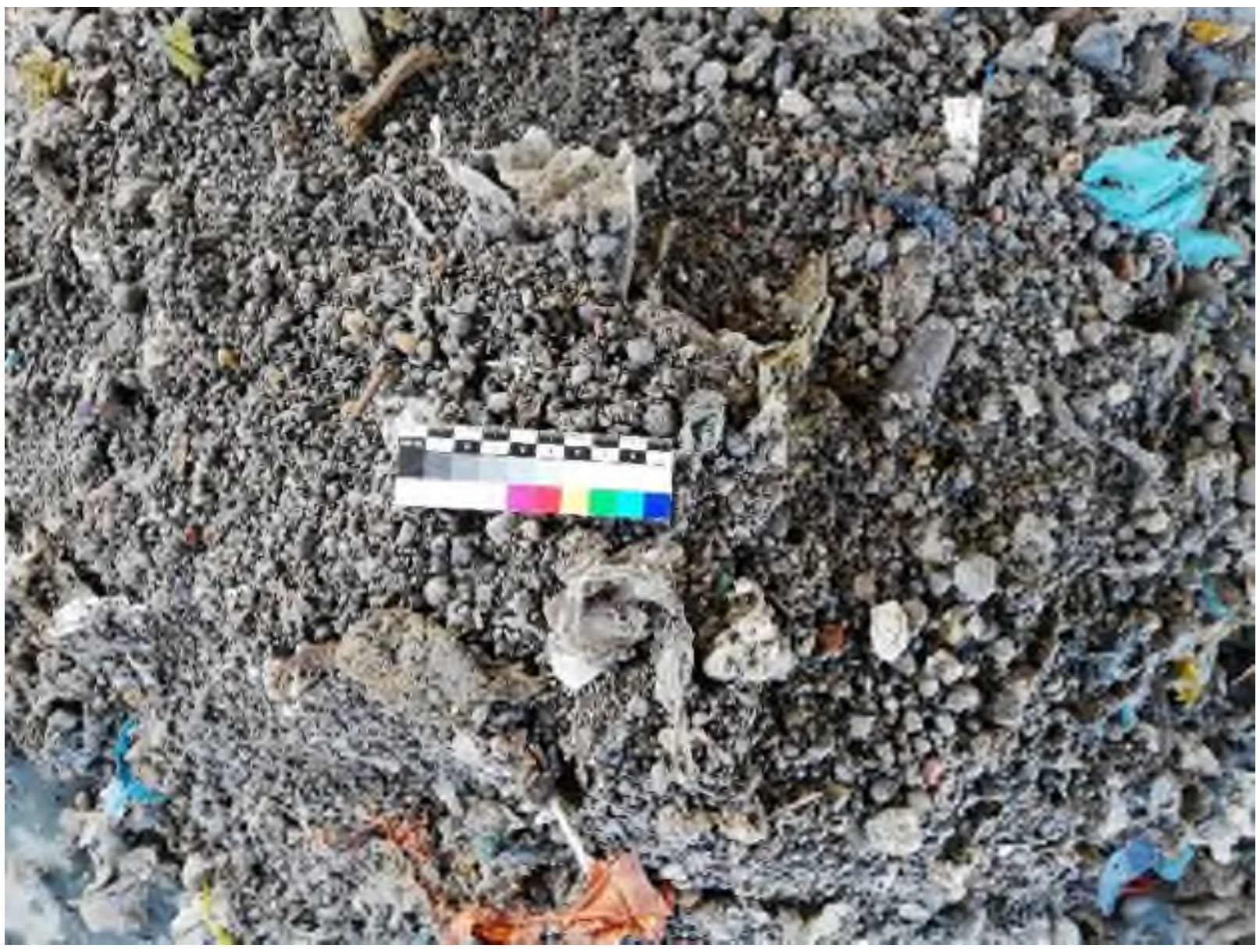

Figure 13: Swimming material from G-IBA (picture taken by Julia Mühl with permission to be used)

3.1.3 Treated mineral fraction

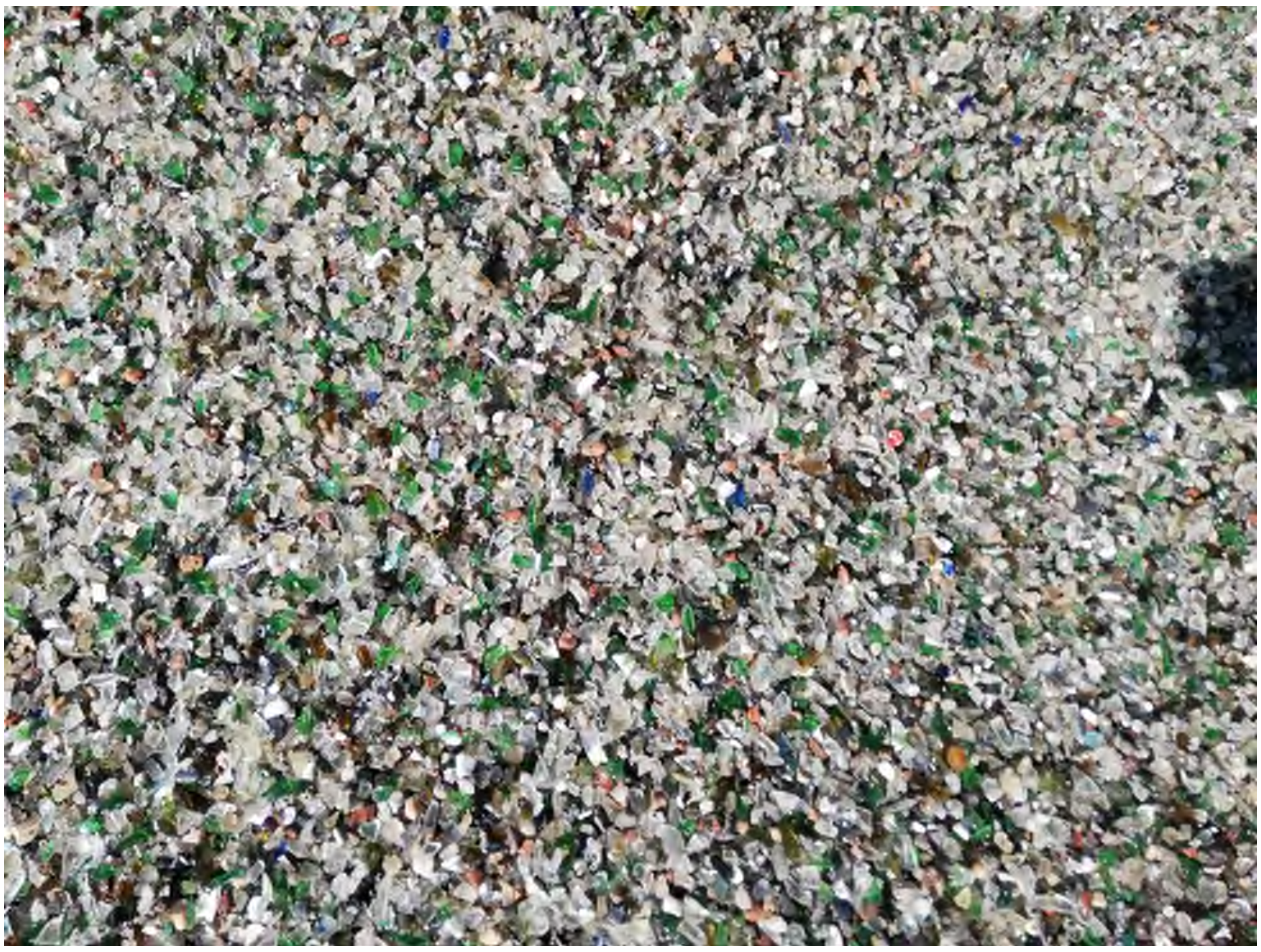

Figure 14: Treated mineral fraction from FB-IBA (fresh/wet material) (picture taken by Julia Mühl with permission to be used)

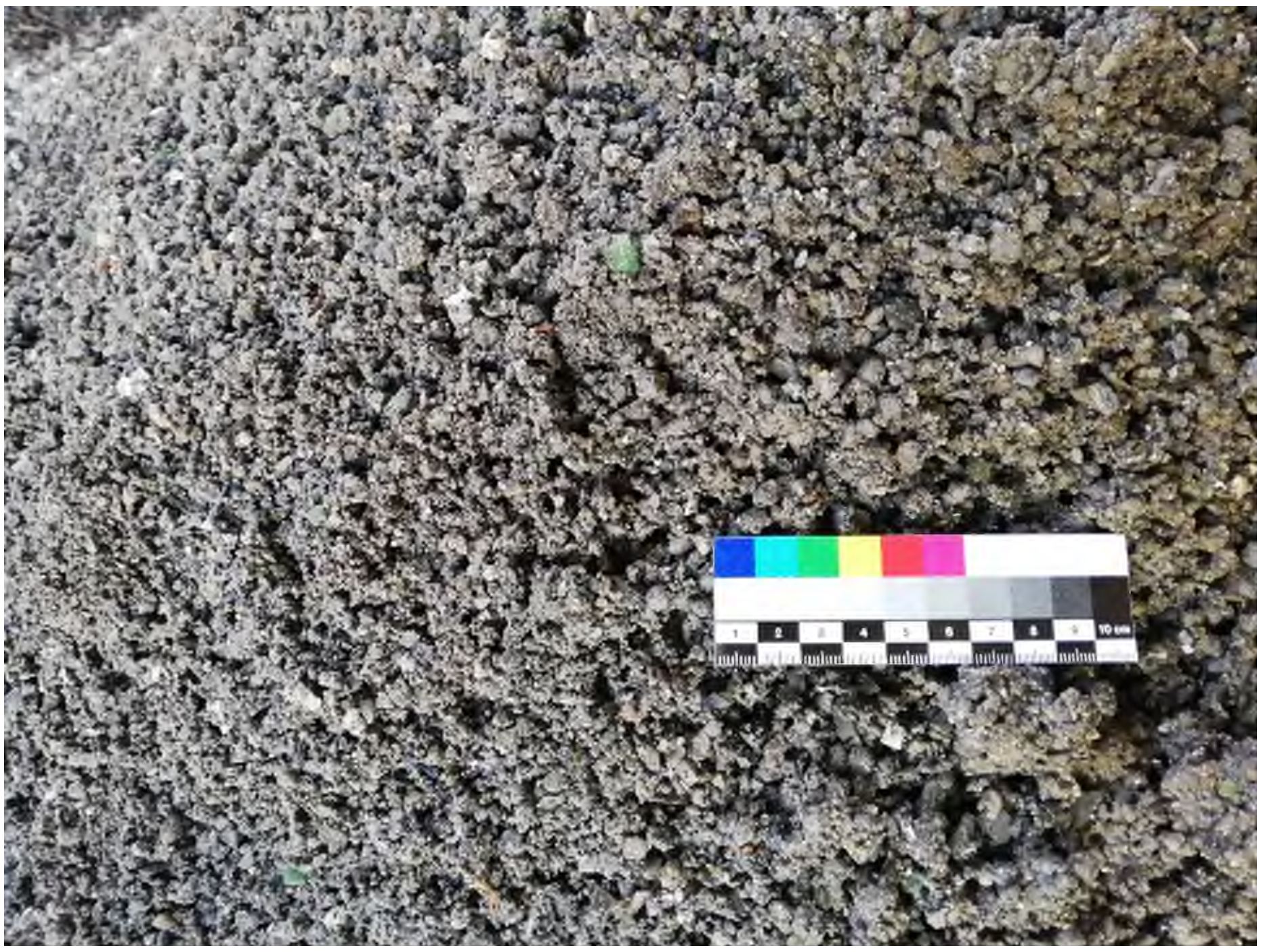

Figure 15: Treated mineral fraction from G-IBA (fresh/wet material) (picture taken by Julia Mühl with permission to be used)

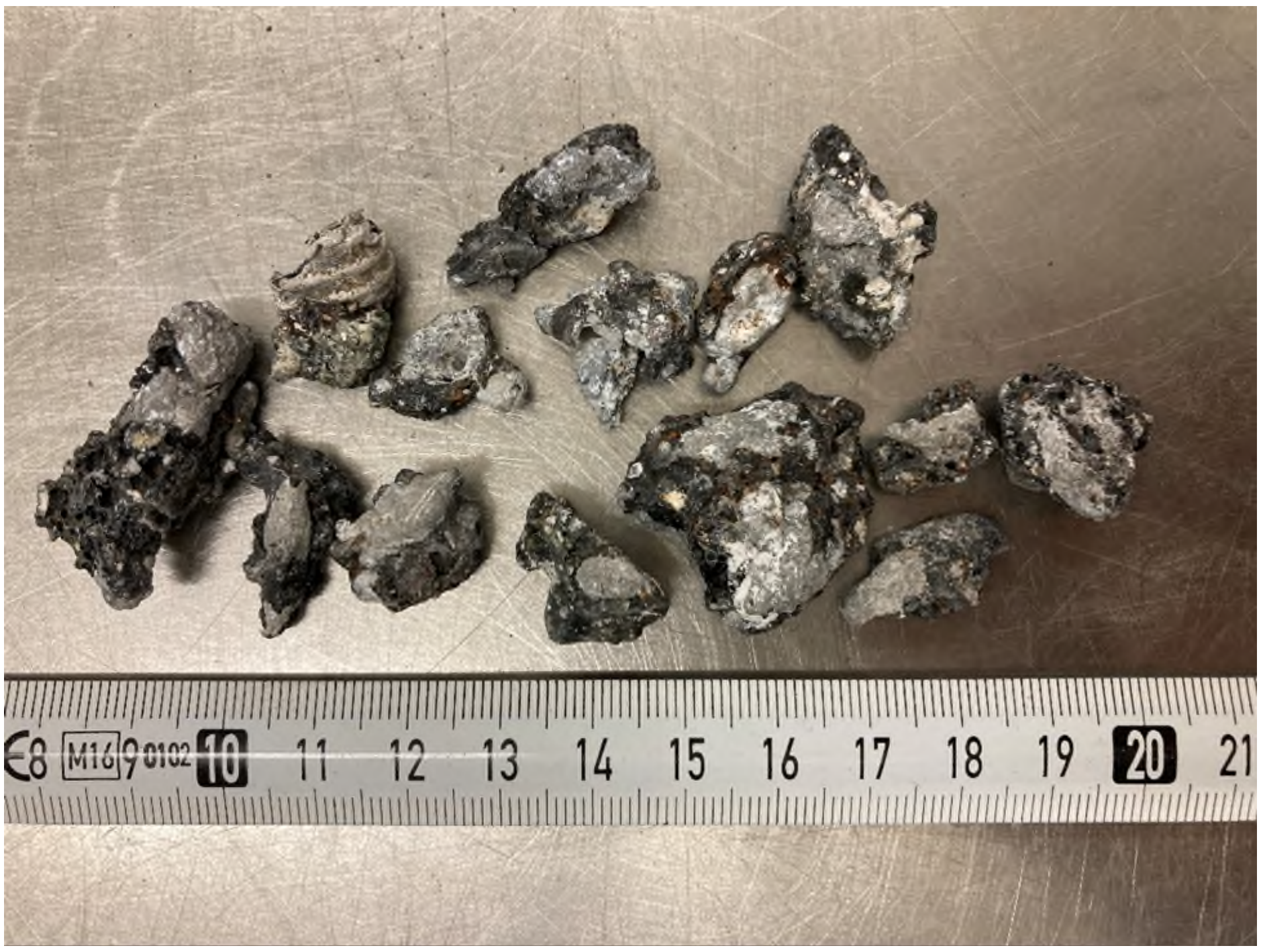

Figure 16: Hard-to-recover aluminium particles (grey stains = aluminium) (own picture)

$$c_{Al,mineral\ fraction} = c_{Al,\ >4mm} \cdot \omega_{>4mm\ of\ mineral\ fraction} + c_{Al,<4mm} \cdot \omega_{Al,<4mm\ of\ mineral\ fraction} \quad (5)$$

Table 8: Mass fraction and content of metallic aluminum of treated mineral fraction

	$\omega_{>4mm}$	$\omega_{<4mm}$	$c_{Al,\ >4mm}$	$c_{Al,mineral\ fraction}$	$c_{Al,<4mm}$
treated mineral fraction G-IBA	29%	71%	0.18%	1.0%	1.3%
treated mineral fraction FB-IBA	28%	72%	0.12%	0.5%	0.6%
Reference	Mühl et al. (2024)			This study	calculated

4 Limitations

No supplementary material

5 Conclusion

No supplementary material

6 References

Blasenbauer, D., Huber, F., Mühl, J., Fellner, J., & Lederer, J. (2023). Comparing the quantity and quality of glass, metals, and minerals present in waste incineration bottom ashes from a fluidized bed and a grate incinerator. *Waste Management*, *161*, 142-155. https://doi.org/10.1016/j.wasman.2023.02.021

Bunge, R., & Bunge, K. (1999). Probenahme auf Altlasten: Minimal notwendige Probenmasse. *Altlasten spektrum*, *3/99*, 174-179.

Esbensen, K. H. (2019). Materials Properties: Heterogeneity and Appropriate Sampling Modes. *Journal of AOAC INTERNATIONAL*, *98*(2), 269-274. https://doi.org/10.5740/jaoacint.14-234

Gerlach, R., & Nocerino, J. (2003). *Guidance for Obtaining Representative Laboratory Analytical Subsamples from Particulate Laboratory Samples*. https://nepis.epa.gov/Exe/ZyPURL.cgi?Dockey=2000GTWM.txt

Gy, P. M. (1998). Sampling for analytical purposes.

Huber, F., Blasenbauer, D., Aschenbrenner, P., & Fellner, J. (2020). Complete determination of the material composition of municipal solid waste incineration bottom ash. *Waste Management*, *102*, 677-685. https://doi.org/10.1016/j.wasman.2019.11.036

Mika, S., Mühl, J., Skutan, S., Aschenbrenner, P., Limbeck, A., & Lederer, J. (2025). Substance flows of heavy metals in industrial-scale municipal solid waste incineration bottom ash treatment: A case study from Austria. *Waste Management*, *195*, 240-252. https://doi.org/10.1016/j.wasman.2025.02.011

Mühl, J., Hofer, S., Blasenbauer, D., & Lederer, J. (2024). Recovery of aluminum, magnetic ferrous metals and glass through enhanced industrial-scale treatment of different MSWI bottom ashes. *Waste Management*, *190*, 557-568. https://doi.org/10.1016/j.wasman.2024.10.025

Skutan, S., & Brunner, P. H. (2005). *Stoffbilanzen mechanisch-biologischer Anlagen zur Behandlung von Restmüll (SEMBA)*. https://www.bmk.gv.at/dam/jcr:493bbae5-18c2-4761-ab41-20ab0ea31431/Studie_SEMBA.pdf
